# Gudan Wan attenuates metabolic dysfunction-associated steatohepatitis by modulating autophagy and the Bcl-2/Caspase-3/Caspase-8 apoptotic axis

**DOI:** 10.3389/fcell.2026.1993264

**Published:** 2026-10-22

**Authors:** Shaocong Han, Qiuyi Li, Zhongneng Ding, Huan Yang, Di Du, Qidi Zhou, Jie Yu, Xingxin Yang

**Affiliations:** 1 The First Clinical College, Yunnan University of Chinese Medicine, Kunming, China; 2 College of Pharmaceutical Science, Yunnan University of Chinese Medicine, Kunming, China; 3 Yunnan Key Laboratory of Southern Medicine Utilization, Yunnan University of Chinese Medicine, Kunming, China

**Keywords:** apoptosis, autophagy, Gudan Wan, metabolic dysfunction-associated steatohepatitis, traditional Chinese medicine

## Abstract

**Background:**

Metabolic dysfunction-associated steatohepatitis (MASH) is a leading global cause of end-stage liver disease, with safe and effective therapies still lacking. Gudan Wan (GDW), a traditional Chinese medicine for Gu Dan (damp-heat and food stagnation with middle-jiao dysfunction), shares analogous pathogenesis with MASH, yet its anti-MASH efficacy remains to be elucidated.

**Methods:**

GDW’s therapeutic efficacy and underlying mechanisms via *in vivo* toxicity and pharmacodynamic evaluation, network pharmacology, western blotting, UHPLC-Q-TOF-MS characterization, and molecular docking. A MASH model of mice was induced by high-fat diet and carbon tetrachloride (HFD+CCl_4_).

**Results:**

A 30-day toxicity test revealed no overt toxicity or organic lesions in mice at GDW doses up to 4000 mg/kg. MASH mice, GDW significantly ameliorated hepatic injury, lipid metabolic disorders, pro-inflammatory cytokine release, and fibrosis. Network pharmacology identified 237 intersecting targets, with apoptosis as the core regulatory pathway and CASP 3/8, AKT1, TP53, BCL2, and TNF as core targets. Western blotting confirmed GDW modulated key apoptotic proteins (upregulating Bcl-2, downregulating Caspase-3/8) and corrected autophagic dysfunction via LC3 and p62 modulation. A total of 179 chemical components were characterized by UHPLC-QTOF-MS, of which 19 were found to enter the liver. Molecular docking predicted favorable binding of the major active components to Caspase-3, AKT1, TP53, and TNF-α, but not to Bcl-2 or Caspase-8, suggesting indirect regulation of the latter two by GDW.

**Conclusion:**

GDW exerts its anti-MASH effects by modulating autophagy and the Bcl-2/Caspase-3/Caspase-8 apoptotic axis, with its favorable safety profile supporting its therapeutic potential for further investigation.

## 1 Introduction

Metabolic dysfunction-associated steatohepatitis (MASH) is recognized as a severe progressive form of metabolic dysfunction-associated steatotic liver disease (MASLD), with its core pathological features characterized by hepatic steatosis accompanied by inflammatory injury, hepatocyte ballooning, and variable degrees of fibrosis (Leow et al., 2023; Tong et al., 2024), and it accounts for approximately 20%–30% of patients with MASLD. As the worldwide prevalence of obesity and type 2 diabetes mellitus is elevated, MASH has been recognized as one of the predominant contributors to end-stage liver diseases including liver cirrhosis, hepatic failure and hepatocellular carcinoma (Devasia et al., 2025; Pipitone et al., 2023), thereby imposing a substantial burden on healthcare systems worldwide. Although resmetirom and semaglutide have demonstrated partial benefits in certain patient subgroups, their clinical response rates remain low, and their use is limited by gastrointestinal and cardiovascular risks (Harrison et al., 2024; Petta et al., 2025). Accordingly, a deeper elucidation of the pathogenic mechanisms underlying MASH and the identification of safe and effective therapeutic strategies are of significant scientific importance and clinical value.

Modern medicine recognizes MASH as organ dysfunction induced by long-term fat accumulation, with a highly complex pathogenesis involving lipotoxicity, oxidative stress, mitochondrial dysfunction, apoptosis, and pro-inflammatory cytokine cascades (LeFort et al., 2024; Steinberg et al., 2025). In addition to these pathological processes, nutritional, hormonal, and gut microbiota-related factors also contribute to the progression of MASH (Li et al., 2025a; Walsh et al., 2025). Although no traditional Chinese medicine (TCM) disease name directly corresponds to MASH, its clinical and pathological features place it within the categories of “intercostal pain”, “phlegm syndrome” and “accumulation” (Zhang et al., 2025). TCM theory posits that this condition arises from dietary excess and heavy intake of fatty and sweet foods, leading to middle-jiao dysfunction and internal damp-heat that invades the liver and gallbladder, ultimately progressing to an intertwined state of “damp-heat, toxin, and stasis” (Sun and Dong, 2024; Zhang et al., 2020). TCM has a long history and unique advantages in the treatment of liver diseases. The characteristics of multi-component, multi-target and holistic regulation of TCM are highly consistent with the complex pathological mechanisms of MASH. Gudan Wan (GDW), a traditional Chinese medicinal formula based on classical TCM theories, is composed of Sophorae Flavescentis Radix (SFR), Gentianae Radix et Rhizoma (GERR), Gardeniae Fructus (GF), Ginseng Radix et Rhizoma (GIRR) and Suis Fellis Pulvis (SFP) at a ratio of 100:33:17:1:2.5. It is mainly applied for the treatment of “Gu Dan” caused by damp-heat and food stagnation (Wang et al., 2022). In this formula, SFR exerts actions of clearing heat, drying dampness, dispersing stasis, and eliminating toxins. GERR and GF purge damp-heat from the liver and gallbladder, while GIRR invigorates the spleen and replenishes qi to protect the middle jiao. Concurrently, SFP promotes defecation and drains pathogenic fire. A combination of cold and warm medicinal properties is adopted in the whole formula, by which both the root pathogenesis and superficial symptoms of the disease are addressed, the effects of clearing heat, draining dampness, resolving stasis and clearing toxin are exerted, and the core pathogenesis of MASH is precisely matched. From a modern pathological perspective, the intertwined pathological state of “damp-heat, toxin and stasis” in TCM may correspond to the complex interplay of lipotoxicity, inflammatory cytokine cascades, oxidative stress and apoptotic signaling in MASH. Therefore, the multi-pronged therapeutic actions of GDW are theoretically aligned with the multi-target regulation required for MASH intervention.

Accumulating pharmacological evidence supports that the active ingredients of the five herbs in GDW have protective effects on MASLD and MASH. Matrine and oxymatrine, core alkaloids of SFR, alleviate MASH by modulating endoplasmic reticulum stress, mitochondrial dysfunction (Gao et al., 2018), and the Sirtuin 1/AMP-activated protein kinase/Liver X receptor/Perilipin 2/Sterol regulatory element-binding protein 1c axes (Xiong et al., 2025). Iridoid glycosides from GERR, including swertiamarin, gentiopicroside, and sweroside, protect against MASH by regulating inflammatory signaling pathways such as p38 Mitogen-activated protein kinase/Nuclear factor kappa B (NF-κB) (Xu et al., 2021), peroxisome proliferator-activated receptor α (PPARα) (Huang et al., 2024), and NLR family pyrin domain containing 3 inflammasome (Yang et al., 2020). Geniposide and genipin, characteristic ingredients of GF, improve glucolipid metabolism and hepatic fibrosis by inhibiting CCCTC-binding factor/Dipeptidyl peptidase 4 (Shen et al., 2021) and Uncoupling protein 2-related pyroptosis (Zhong et al., 2018). Ginsenosides from GIRR, including ginsenoside Rg1 and Re, attenuate MASH pathology through regulation of the miR-375–3p/Autophagy-related protein 2B/Phosphatase and tensin homolog-AKT axis (Chen et al., 2023) and the Phosphoinositide 3-kinase (PI3K)/AKT/Toll-like receptor 4/NF-κB signaling pathways (Zhang et al., 2024). Bile acids from SFP, including hyodeoxycholic acid and tauroursodeoxycholic acid, ameliorate MASLD by suppressing Ras-related nuclear protein-mediated nucleocytoplasmic shuttling of PPARα (Zhong et al., 2023) and remodeling gut microbiota and bile acid homeostasis (Wang et al., 2024). Although each constituent herb of GDW has been individually reported to exert protective effects against MASLD/MASH, whether and how the complete formula attenuates MASH at the molecular level, and which components actually reach the liver to mediate this effect, have not been systematically established.

Thus, in this study, the *in vivo* safety of GDW was evaluated via a 30-day repeated-dose toxicity test, and its anti-MASH efficacy was subsequently verified in a MASH mouse model induced by a HFD combined with CCl_4_. On this basis, potential action targets and related signaling pathways of the active components in GDW were systematically predicted via network pharmacology, and the key pathways and targets were further verified in animal experiments. Meanwhile, Ultra-high-performance liquid chromatography coupled with quadrupole time-of-flight mass spectrometry (UHPLC-QTOF-MS)-based component identification, molecular docking and *in vitro* experiments collectively verified the hepatoprotective effects of core bioactive constituents against free fatty acids (FFA)-induced injury in L02 cells. This study was designed to systematically elucidate the molecular mechanism of GDW in the treatment of MASH through the aforementioned multi-level experiments, and provide scientific basis and experimental support for the development of innovative therapeutic agents for MASH.

## 2 Materials and methods

### 2.1 Reagents and antibodies

Resmetirom (purity ≥98%, Cat. NO. BD764733-1g) was purchased from Bidepharm (Shanghai, China). Carbon tetrachloride (CCl_4_, Cat. NO. RH694624) was obtained from Jskchem (Shanghai, China). Bovine serum albumin (BSA, Cat. No. 4240GR100) was obtained from BioFroxx GmbH (Einhausen, Germany). Lipopolysaccharides (LPS, Cat. No. L8880) was purchased from Solarbio Co., Ltd (Shanghai, China). Oleate (S817542) was obtained from Macklin Biochemical Co., Ltd (Shanghai, China) and palmitate (Cat. No. S70021-5G) was purchased from Beijing Psaitong Biotechnology Co., Ltd (Beijing, China). Dulbecco’s Modified Eagle Medium, penicillin/streptomycin, and trypsin were purchased from Gibco (Waltham, MA, United States of America). Fetal bovine serum (FBS) was purchased from Procell Life Science & Technology Co., Ltd (Wuhan, China). Phosphate-buffered saline (PBS) was purchased from Biosharp Life Sciences Co., Ltd (Hefei, China). The hematoxylin and eosin (H&E) stain kit, oil red O stain kit, RIPA lysis buffer, and 5x loading buffer were purchased from Solarbio Co., Ltd (Shanghai, China). Kits to measure Triglycerides (TG, Cat. No. A110-1-1), Total Cholesterol (TC, Cat. No. A111-1-1), Alanine Aminotransferase (ALT, Cat. No. C009-2-1), Aspartate Aminotransferase (AST, Cat. No. C010-2-1), Low-Density Lipoprotein Cholesterol (LDL-C, Cat. No. A113-1-1), High-Density Lipoprotein Cholesterol (HDL-C, Cat. No. A112-1-1), Alkaline phosphatase (ALP, Cat. No. A059-2-2), Total Protein (TP, Cat. No. A045-2-2), Albumin (ALB, Cat. No. A028-2-1), Blood Urea Nitrogen (BUN, Cat. No. C013-2-1), Creatinine (CREA, Cat. No. C011-2-1) and Blood Glucose (GLU, Cat. No. A154-1-1) were purchased from Jiancheng Bioengineering Institute (Nanjing, China). Primary antibodies against LC3 (Cat.4108), p62 (Cat.18420-1-AP), and GAPDH (Cat.81640-5-RR) were purchased from Cell Signaling Technology (Danvers, MA, United States of America). Primary antibodies against B-cell lymphoma 2 (Bcl-2) (Cat. 66799-1-Ig) and Horseradish peroxidase (HRP)-conjugated secondary antibodies were purchased from Proteintech Group, Inc. (Wuhan, China). Caspase-3 (Cat.341034) and Caspase-8 (Cat.250106) were purchased from Chengdu Zen Bioscience Co., Ltd (Chengdu, China). Enhanced chemiluminescence (ECL) Detection Kit (Cat. No. AC0454) were purchased from Changsha Cinotohi Technology Co., Ltd (Changsha, China). HFD (40% kcal% fat, 20% kcal% fructose, Cat. No. D18402002/D09100310) was purchased from SYSE Biotech. Co., Ltd (Changzhou, China). Basal feed was purchased from SiPeiFu Biotech. Co., Ltd (Beijing, China). Additionally, 4% paraformaldehyde and electron microscope fixative were purchased from Servicebio Biotech Co., Ltd (Wuhan, China).

### 2.2 Preparation of GDW extract

The dried medicinal materials of SFR, GERR, GF, GIRR, and SFP were weighed and mixed at a ratio of 100:33:17:1:2.5 (w/w). The mixture was decocted twice with distilled water at a solvent-to-material ratio of 10:1 (v/w), each for 1 h. After filtration to remove insoluble residues, the combined filtrates were concentrated under reduced pressure and lyophilized to obtain the aqueous extract powder of GDW, with a yield of 25.2% (w/w).

### 2.3 *In vivo* experiments

#### 2.3.1 Animals and experimental protocol

66 specific pathogen-free (SPF) C57BL/6J mice, aged 8 weeks and weighing 21 ± 1 g, were obtained from Beijing SPF Biotechnology Co., Ltd (Laboratory Animal Quality Certificate No.: 110324251105382046; Production License No.: SCXK (jing-2024-0001). The mice were randomly allocated to experimental groups and the corresponding treatment group. To ensure blinding, group assignment and drug administration were carried out by separate investigators, thereby minimizing potential bias. All animals were maintained in the Animal Experiment Center of Yunnan University of Chinese Medicine under standard conditions. All experimental procedures were reviewed and approved by the Institutional Animal Ethics Committee of the same university (Approval No: R-062023151).

##### 2.3.1.1 30-day toxicity study

30 male C57BL/6J mice were randomly assigned into three groups (n = 10 per group): a vehicle control group, a low-dose GDW group (1,000 mg/kg), and a high-dose GDW group (4,000 mg/kg), corresponding to approximately 2-fold and 8-fold the equivalent clinical daily dose for adults, respectively. GDW was administered by oral gavage once daily at a dosing volume of 0.1 mL/10 g body weight for 30 consecutive days. Mice in the vehicle control group received an equal volume of normal saline following the same administration schedule. During the treatment period, the general condition of the mice was monitored daily, including appearance, respiration, spontaneous activity, food and water intake, nasal and oral secretions, urine and feces, as well as mortality. Body weight was recorded throughout the experiment.

##### 2.3.1.2 MASH model establishment and therapeutic intervention

After a 7-day acclimation period, C57BL/6J mice were randomly allocated to six groups (n = 6/group). All mice except the Control group were fed a HFD. From week 13 of HFD feeding, weekly intraperitoneal CCl_4_ (0.05 mL/kg) injections were administered, along with daily oral gavage administration dosing of the assigned treatments: saline for Control and Model, resmetirom (3.12 mg/kg/d) for positive control, and GDW at 4000 mg/kg/d (GDW-H), 1000 mg/kg/d (GDW-M) or 500 mg/kg/d (GDW-L). Weekly body weight and food intake were recorded.

After the experiment, mice were fasted for 12 h, anesthetized with 2% pentobarbital sodium, and exsanguinated via abdominal aorta. Blood samples were clotted at room temperature for 4 h, centrifuged at 3,500 rpm for 15 min at 4 °C, and serum was stored at -80 °C. The major organs (heart, liver, spleen, lungs, kidneys, and brain) were then excised, cleaned of adherent fat and connective tissue, and weighed on an analytical balance (0.1 mg precision). Organ coefficients were calculated as (organ weight /body weight) × 100.

#### 2.3.2 Histological and pathological examination of liver tissue

##### 2.3.2.1 Oil red O staining

Liver tissues from the left lobe were embedded in optimal cutting temperature compound, frozen at -20 °C for 30 min, and sectioned at 8 µm on a cryostat. Sections were mounted on slides, soaked in 60% isopropanol for 5 s, stained with Oil red O in 60% isopropanol for 15 min, washed with PBS, and counterstained with hematoxylin for 10 s. After mounting with glycerol gelatin, the sections were visualized using a NIKON DS-U3 imaging system (Nikon, Tokyo, Japan).

##### 2.3.2.2 H&E staining

Liver tissues fixed in 4% paraformaldehyde for 24 h were dehydrated in graded ethanol, cleared with xylene, and embedded in paraffin. Serial sections (4 µm) were cut using a microtome, deparaffinized, and subjected to hematoxylin and eosin (H&E) staining. Following hematoxylin staining, sections were differentiated, blued, and washed. They were then dehydrated in 95% ethanol for 1 min, counterstained with eosin for 15 s, further dehydrated, and mounted with neutral resin for light microscopic examination.

##### 2.3.2.3 Masson staining

Masson staining was conducted on paraffin-embedded liver sections. Sections were stained with hematoxylin for 2 min, rinsed in distilled water, differentiated in 1% acid alcohol until nuclei appeared gray-black, and blued. After staining with ponceau-acid fuchsin, excess dye was removed with 0.2% acetic acid. Sections were differentiated in phosphomolybdic acid until collagen turned light red, rinsed, and stained with aniline blue to highlight collagen fibers. Excess aniline blue was washed with 0.2% acetic acid. The sections were then dehydrated, cleared, mounted with neutral resin, and examined under a light microscope.

##### 2.3.2.4 Transmission electron microscope (TEM)

Liver tissues were fixed in electron microscopy fixative at 4 °C for 2-4 h, rinsed in 0.1 M phosphate buffer (pH 7.4), post-fixed in 1% osmium tetroxide at room temperature for 2 h, and rinsed again. After dehydration in a graded ethanol-acetone series (50-100%, 15 min each), samples were infiltrated with acetone/Epoxy Resin 812 mixtures (1:1 and 2:1) and pure resin, embedded in molds, and polymerized at 37 °C overnight and then at 60 °C for 48 h. Ultrathin sections (60-80 nm) were cut, double-stained with uranyl acetate and lead citrate (15 min each), air-dried, and examined by TEM, images were acquired for analysis.

##### 2.3.3 Determination of biochemical parameters in serum and liver tissues

Serum TC, TG, ALT, AST, HDL-C, LDL-C, ALP, TP, ALB, BUN, CREA and GLU were measured using commercial kits after thawing samples on ice. For liver tissue analysis, approximately 0.1 g of thawed tissue was homogenized in 0.9 mL of pre-chilled saline with grinding beads using a tissue grinder at 4 °C and 60 Hz for 4 cycles. The homogenates were centrifuged at 10,000 rpm for 10 min at 4 °C, and the supernatants were collected for biochemical measurements. Hepatic TC, TG, ALT, AST, HDL-C, and LDL-C were determined using the commercial kits. All procedures performed according to the manufacturers instructions. The absorbance values were measured using a SpectraMax Plus 384 microplate reader (Molecular Devices, Sunnyvale, CA, United States of America).

#### 2.4 Network pharmacology prediction and molecular docking

Potential bioactive compounds in GDW and their molecular targets were screened using the Traditional Chinese Medicine Systems Pharmacology Database and Analysis Platform (TCMSP, https://tcmspw.com/tcmsp.php), with Oral bioavailability ≥30% and Drug-likeness ≥0.18 as the selection criteria. The identified bioactive compounds were further validated using the SymMap database (http://www.symmap.org/) (Du et al., 2022; Yu et al., 2025). MASH-associated targets were retrieved from GeneCards (https://www.genecards.org). Overlapping targets between GDW and MASH were identified by a Venn diagram generated via the Venny tool. A protein-protein interaction (PPI) network was constructed using the STRING database (https://cn.string-db.org/) and visualized with Cytoscape 3.7.0. Kyoto Encyclopedia of Genes and Genomes (KEGG) pathway enrichment analysis was conducted using the DAVID platform (https://david.ncifcrf.gov). The structures of candidate compounds were retrieved from PubChem (https://pubchem.ncbi.nlm.nih.gov/). The target protein sequences were obtained from UniProt (https://www.uniprot.org/uniprotkb), and the corresponding crystal structures were downloaded from the Protein Data Bank (https://www.rcsb.org/). Receptor proteins were processed using PyMOL (version 3.0.3) and AutoDockTools (version 1.5.7). Molecular docking was performed using AutoDock Vina (version 1.2.7), and binding affinities were calculated. Docking results were visualized using PyMOL.

#### 2.5 Western blot

Western blotting was performed on liver tissue prepared in RIPA buffer containing protease inhibitors. Protein samples (20 μg) were separated by 10% SDS-PAGE and transferred onto PVDF membranes. Membranes were blocked with 5% defatted milk powder in TBST and incubated with primary antibodies, followed by HRP-conjugated secondary antibodies. Protein bands were imaged by using an ECL Detection Kit and the OI capture imaging system (Bio-oi, Guangzhou, China). Band intensities were quantified using ImageJ software (version 1.54f) and normalized to GAPDH.

#### 2.6 Identification of GDW components in the liver of MASH mice

##### 2.6.1 Sample preparation

Liver tissue samples from the control group and the GDW-treated group were thawed at 4 °C. Approximately 0.2 g of liver tissue from each sample was transferred into a homogenization tube, minced into small pieces using scissors, and homogenized with 1.0 mL of physiological saline in an ice bath. The homogenate was transferred to a 5 mL centrifuge tube and mixed with 3.0 mL of acetonitrile by vortexing for 30 s, followed by incubation at 4 °C for 20 min. The samples were centrifuged at 12,000 rpm for 15 min at 4 °C. The supernatants were collected and evaporated to dryness under a gentle stream of nitrogen. The dried residues from all individual samples within the same experimental group were pooled into a single centrifuge tube and reconstituted in 200 μL of methanol. After vortex-mixing for 5 s, the solution was ultrasonicated for 20 min and centrifuged at 12,000 rpm for 10 min at 4 °C. The resulting supernatant was collected and subjected to chromatographic analysis.

In addition, the GDW drug extract solution (1 mg/mL) was subjected to the same sample preparation procedure as described above and subsequently analyzed under identical LC-MS/MS conditions.

##### 2.6.2 LC-MS/MS analyses

LC-MS/MS analyses were executed on a Thermo UHPLC-Q Exactive system (Thermo Scientific, Waltham, MA, United States of America) and an ACQUITYHSS T3 column (1.8 μm, 100 mm × 2.1 mm, Waters, Waltham, MA, United States of America). The mobile phase consisted of (A) acetonitrile and (B) 0.1% formic acid in water. Gradient elution was carried out as follows: 0–3 min, 3% A; 3–8 min, 3%–20% A; 8–23 min, 20%–45% A; 23–33 min, 45%–65% A; 33–35 min, 65%–100% A and 35–40 min, 100% A. The sample injection volume was 3 μL, with a flow rate of 0.4 mL/min. Mass spectral signals of the samples were acquired in both positive and negative ion scanning modes using a Thermo UHPLC-Q Exactive Mass Spectrometer equipped with an electrospray ionization (ESI) source, and the scan mass range was 70–1050 m/z. The spray voltage was 3500 V for the positive ion mode and 2800 V for the negative ion mode. The sheath gas flow rate was 40 psi and the auxiliary gas flow rate was 10 psi. The collision energy was set to a cyclic collision energy of 20-40-60 V.

#### 2.7 *In vitro* experiments

L02 cells were plated in 6-well plates at 3 × 10^5^ cells/well and cultured for 48 h to allow attachment. Subsequently, they were exposed to 0.75 mM FFA (oleate: palmitate ratio of 2:1) and 10 μg/mL LPS for 24 h to induce steatohepatitis. After model establishment, cells were treated for 24 h with resmetirom (100 μM), anagyrine, lupanine, matrine, oxysophocarpine, sophocarpine, and taurohyocholic acid (500 μM or 1 mM), with untreated cells serving as controls. Cells were then harvested for further analysis. Measurement and determination of TC, TG, ALT, AST, HDL-C, LDL-C, TNF-α, IL-6 and IL-1β content in cell lysate using commercial assay kits.

#### 2.8 Data availability and statistical analysis

Data were analyzed using GraphPad Prism 10.1.2 and presented as mean ± standard deviation (S.D). One-way analysis of variance (ANOVA) was used for multiple group comparisons. Statistical significance was defined as *P < 0.05, **P < 0.01, ***P < 0.001, ****P < 0.0001.

## 3 Results

### 3.1 Long-term toxic effects of GDW in mice after 30 days of continuous administration

The results of the 30-day toxicity test showed that during the administration period, the mice in all groups exhibited glossy fur, normal behavioral activities, food intake, and defecation, with no signs of toxicity or mortality observed. No significant differences in body weight were found among the mice at each time point (Figure 1A). The organ index (organ weight/body weight) results indicated that no statistically significant differences were detected between each GDW dose group and the control group (Figure 1B).

**FIGURE 1:**
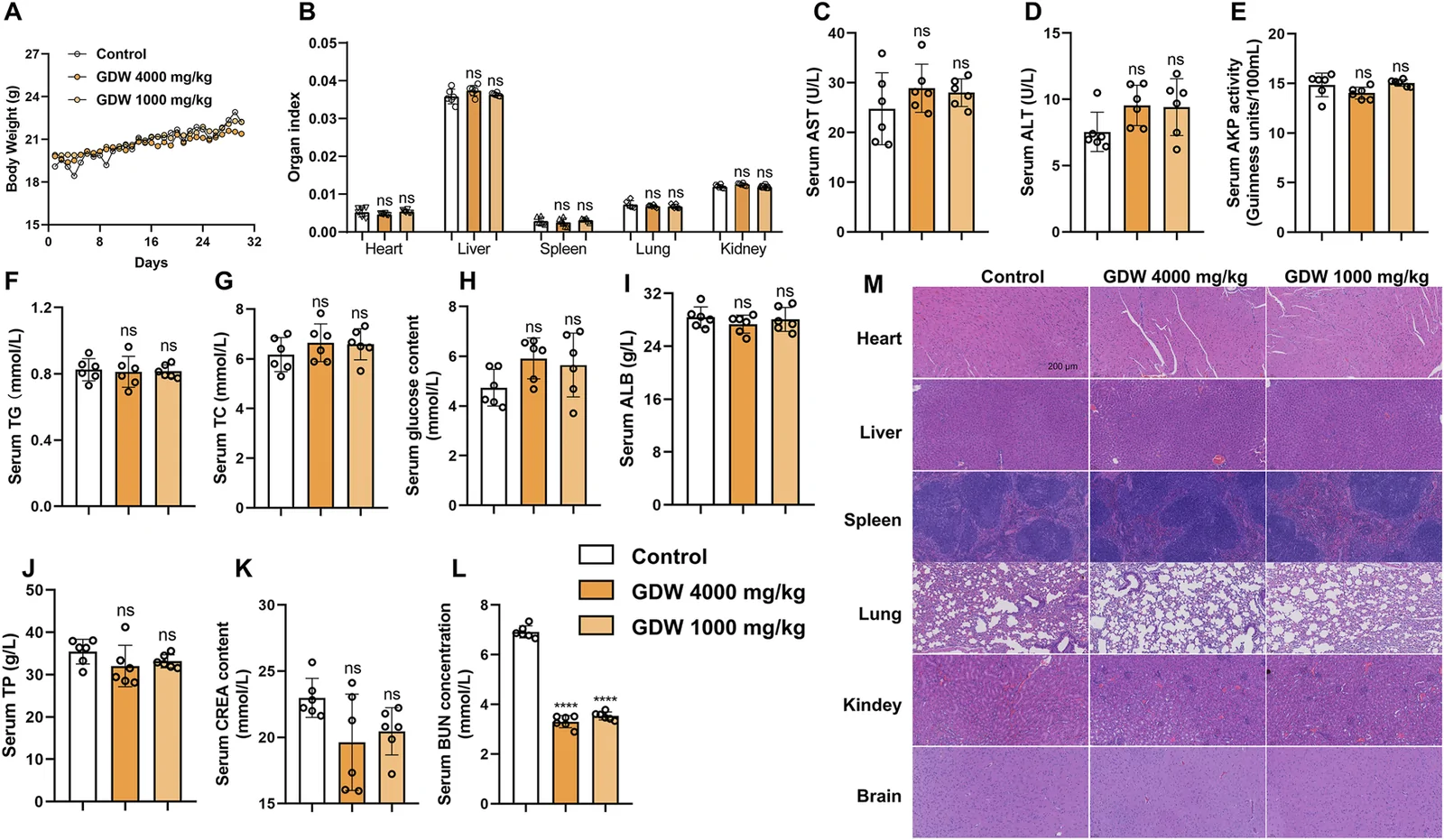
Long-term toxic effects of GDW in mice after 30 days of continuous administration. **(A)** Body weight change curves of mice in all groups during the experimental period; **(B)** Organ indices of the heart, liver, spleen, lungs, and kidneys; **(C,D)** serum AST and ALT levels; **(E)** serum AKP activity; **(F–H)** serum TG, TC, and glucose concentrations; **(I,J)** serum ALB and TP levels; **(K,L)** serum CREA and BUN concentrations; **(M)** H&E stained histological sections of the heart, liver, spleen, lungs, kidneys, and brain. Groups: Control, normal control; GDW 1000 mg/kg, low-dose; GDW 4000 mg/kg, high-dose. ns, no significant difference, ****P < 0.0001 vs. the control group.

A total of 10 serum biochemical parameters, encompassing liver function (ALT, AST, AKP), renal function (BUN, CREA), and metabolism-related indicators (TC, TG, GLU), were measured (Figures 1C–K). The results demonstrated that, compared with the control group, serum BUN levels were significantly decreased in the low- and high-dose GDW groups (Figure 1L). The remaining biochemical indicators (ALT, AST, AKP, CREA, TC, TG, and GLU) showed no significant differences between any GDW dose group and the control group (Figures 1C–K).

H&E staining further confirmed that, relative to the control group, no obvious structural abnormalities, inflammatory cell infiltration, cellular degeneration, or necrosis were observed in the heart, liver, spleen, lung, kidney, or brain tissues of mice in each GDW dose group (Figure 1M).

In conclusion, under the present experimental conditions, continuous intragastric administration of GDW at each dose (1000, 4000 mg/kg) for 30 days did not produce obvious toxic reactions in mice, suggesting that it exhibits a favorable safety profile.

### 3.2 GDW improves liver injury and histopathology in MASH mice

Next, the therapeutic potential of GDW against MASH was investigated. In the MASH model group, hepatic enlargement and marked fat accumulation were observed (Figure 2A), accompanied by significantly increased body weight and liver index (Figures 2B,C), and serum and liver ALT and AST levels were significantly elevated, indicating extensive hepatocyte injury (Figures 2D–G). After GDW intervention, the appearance of the liver was ameliorated, a decreasing trend in the liver index was observed in the middle and high dose groups, and ALT and AST levels were decreased. H&E staining pathological results further confirmed diffuse hepatocyte fatty vacuolar degeneration and pronounced inflammatory infiltration in the model group. In contrast, the degree of hepatic steatosis and inflammatory infiltration was alleviated in the GDW treatment groups, and the hepatic pathological structure was restored (Figure 2H). Collectively, these results suggest that GDW attenuates hepatic pathological changes in MASH, reduces liver injury, and alleviates hepatocyte steatosis, thereby exerting hepatoprotective effects.

**FIGURE 2:**
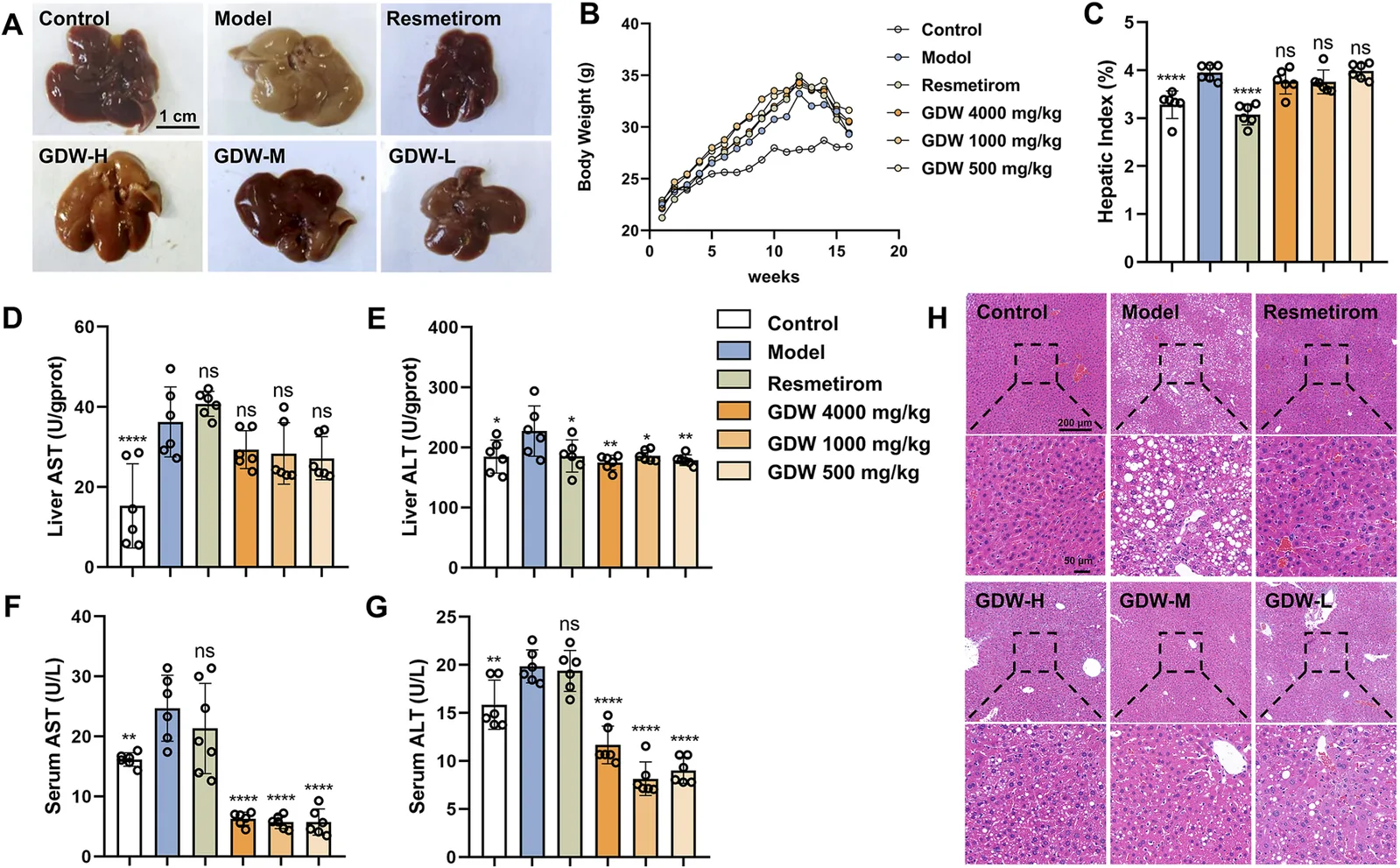
GDW alleviates hepatic injury and histopathological lesions in MASH mice. **(A)** The appearance of the liver in each group, scale bar = 1 cm. **(B)** Body weight change curves of mice in all groups during the experimental period. **(C)** Hepatic index of mice. Hepatic levels of **(D)** AST, **(E)** ALT, and Serum levels of **(F)** AST, **(G)** ALT. **(H)** Representative hematoxylin and eosin **(H, E)** staining of liver sections. Scale bar, 200 μm (up), 50 μm (bottom). Control, normal control group; Model, model group; Resmetirom, positive drug control group; GDW-H, M, L, GDW at doses of 4000 mg/kg, 1000 mg/kg and 500 mg/kg, respectively. ns, no statistically significant difference, * P < 0.05, ** P < 0.01 and **** P < 0.0001 vs. the model group.

### 3.3 GDW ameliorates hepatocellular lipid accumulation in MASH mice

Lipid metabolic disorder is recognized as the earliest pathological feature of MASH. Serum and hepatic lipid indicators, as well as hepatic Oil Red O staining, were examined to observe the intervention effects of GDW on lipid metabolism disorders in MASH model mice. Compared with the control, the levels of serum TC, TG and LDL-C were significantly increased (Figures 3A–C), while serum and hepatic HDL-C was decreased (Figures 3D,H), and hepatic TC, TG, and LDL-C were accumulated in large amounts (Figures 3E–G), indicating significant lipid metabolism disorders. After administration of Resmetirom and different doses of GDW, the abnormal serum and hepatic lipid indicators were significantly improved. By hepatic Oil Red O staining, massive lipid droplet accumulation was observed in hepatocytes of the model group, and the degree of lipid droplet accumulation was markedly alleviated in each treatment group (Figure 3I). The above results demonstrate that GDW could significantly ameliorate hepatic lipid metabolism disorders and reduce hepatocyte lipid deposition in MASH mice.

**FIGURE 3:**
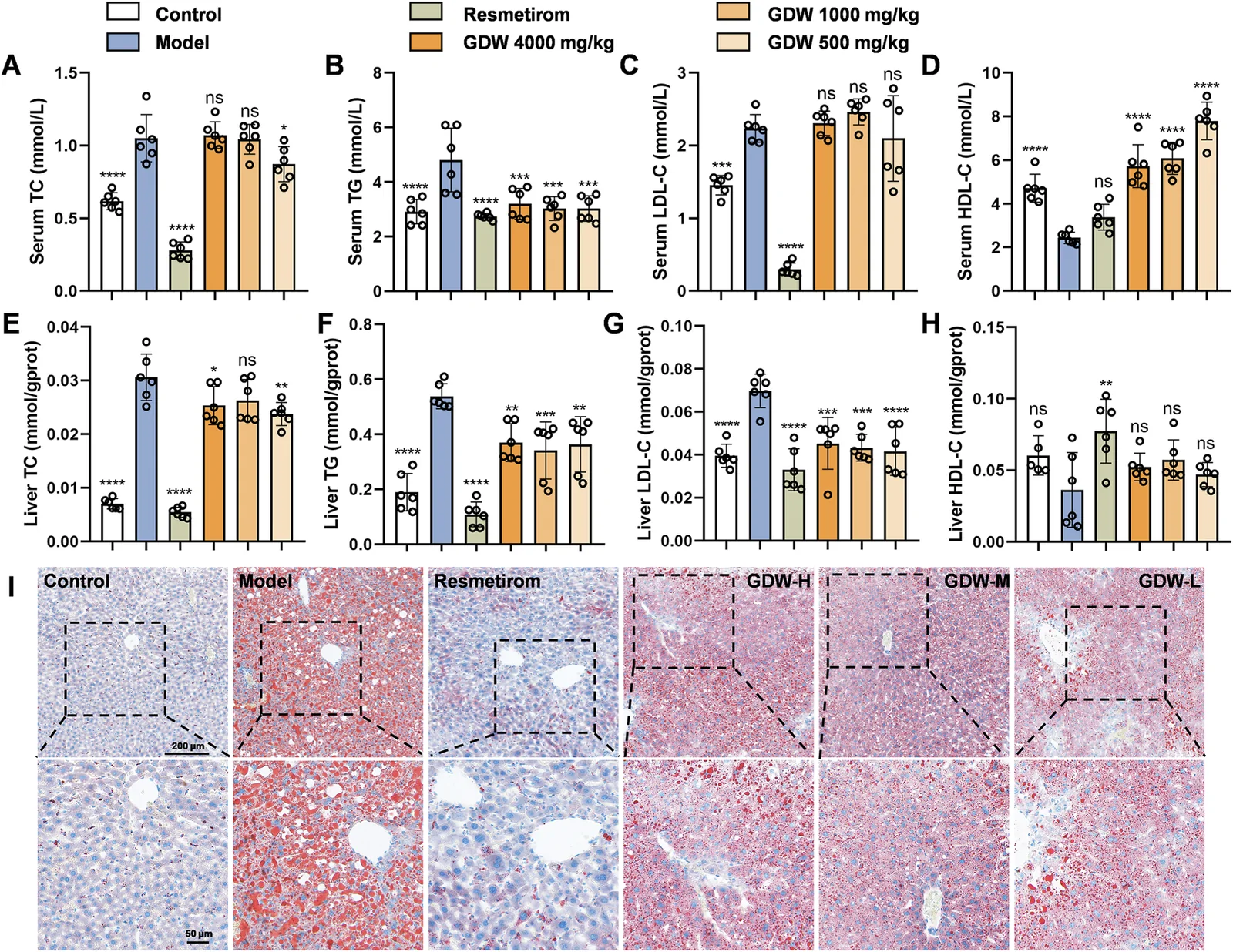
GDW ameliorates hepatocellular lipid accumulation in MASH mice. **(A–D)** Serum TC, TG, LDL-C, and HDL-C levels. **(E–H)** Hepatic TC, TG, LDL-C, and HDL-C levels. **(I)** Representative Oil Red O staining of liver sections, scale bar, 200 μm (up), 50 μm (bottom). Control, normal control group; Model, MASH model group; Resmetirom, positive drug control group; GDW-H, M, L: GDW at doses of 4000 mg/kg, 1000 mg/kg, and 500 mg/kg, respectively. ns, no significant difference, * *P* < 0.05, ** *P* < 0.01, *** *P* < 0.001 and **** *P* < 0.0001 vs. the model group.

### 3.4 GDW effectively mitigates hepatic inflammation and fibrosis in MASH mice

Progressive aggravation of lipid metabolism disorder, hepatic inflammatory infiltration and fibrosis constitute the core pathological processes underlying the initiation and progression of MASH. Abnormal lipid accumulation can further activate hepatic inflammatory responses, induce aberrant collagen deposition in liver tissue, and drive the progression from simple fatty liver to steatohepatitis and liver fibrosis. On the basis of the confirmed effectiveness of GDW in ameliorating lipid metabolism disorders and reducing hepatocyte lipid deposition in MASH mice, serum and hepatic pro-inflammatory cytokine levels were further evaluated, and Masson staining was performed to assess the intervention effects of GDW on inflammation and liver fibrosis in MASH mice.

The results demonstrated that the levels of serum and hepatic pro-inflammatory cytokines, including IL-6, Tumor Necrosis Factor α (TNF-α), and IL-1β were markedly elevated in the model group compared with the control group (Figures 4A–F). Following treatment, inflammatory responses were attenuated to varying degrees across groups. Compared with the model group, Resmetirom significantly reduced serum TNF-α and hepatic IL-1β and IL-6, whereas serum IL-6, serum IL-1β, and hepatic TNF-α showed nonsignificant downward trends. GDW-L significantly decreased serum IL-1β, with nonsignificant reductions in the remaining inflammatory factors. GDW-M showed generally decreasing but nonsignificant trends in all measured serum and hepatic inflammatory factors. GDW-H significantly reduced hepatic TNF-α, while other inflammatory factors tended to decrease without statistical significance (Figures 4A–F).

**FIGURE 4:**
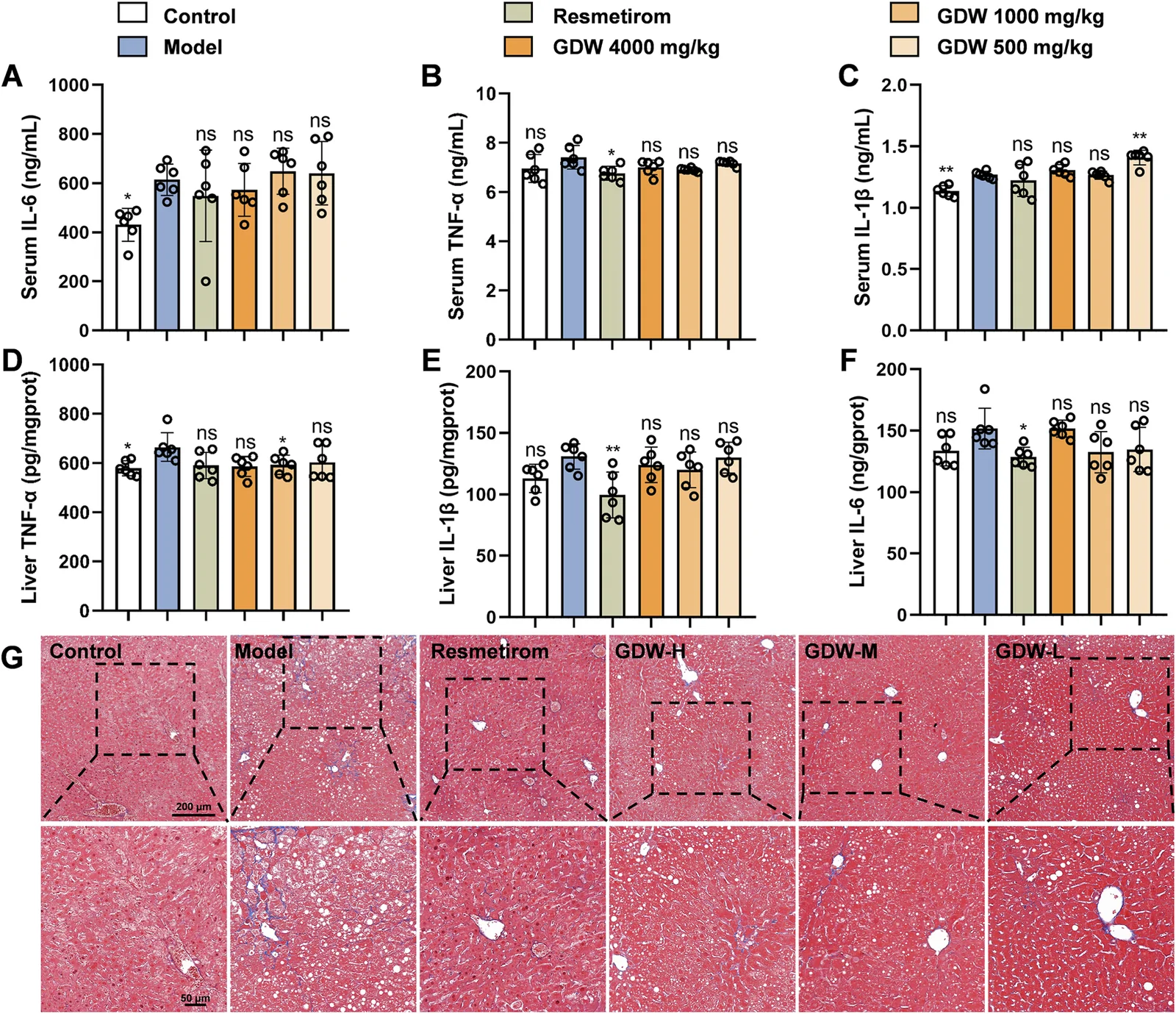
GDW effectively mitigates hepatic inflammatory and fibrosis progression in MASH mice. **(A–C)** Serum levels of IL-6, TNF-α, and IL-1β; **(D–F)** Hepatic levels of IL-6, TNF-α, and IL-1β; **(G)** Representative Masson’s trichrome of liver sections, scale bar, 200 μm (up), 50 μm (bottom). Control, normal control group; Model, MASH model group; Resmetirom, positive drug control group; GDW-H, M, L, GDW at 4000, 1000, and 500 mg/kg, respectively. ns, no significant difference, * *P* < 0.05 and ** *P* < 0.01 vs. the model group.

Masson staining of liver tissues revealed that extensive intrahepatic collagen deposition and severe fibrotic lesions were observed in the model group. The area of blue collagen deposition in the liver was markedly reduced, and the degree of fibrosis was alleviated after administration of GDW (Figure 4G). These findings indicate that systemic and hepatic inflammatory responses were inhibited, and hepatic collagen fiber deposition was relieved, indicating that the progression of hepatic fibrosis was delayed by GDW in MASH model mice.

### 3.5 Apoptotic pathway of GDW against MASH elucidated by network pharmacology

Previous *in vivo* experimental results have confirmed that GDW effectively ameliorated lipid metabolism disorders, inhibits hepatic inflammatory infiltration, and reduces hepatic collagen deposition and fibrotic damage in MASH model mice, thereby exerting a protective effect on the pathological progression of MASH. To further systematically clarify the multi-dimensional targets and molecular mechanisms underlying the intervention of GDW on MASH, a network pharmacology strategy was adopted to explore the potential mechanism of GDW against MASH.

Firstly, the active ingredient targets of the formula and MASH related disease targets were screened, and a herb-ingredient-target-disease interaction network was constructed to intuitively characterize the multi-component and multi-target regulatory characteristics of formula. A total of 100 pharmacologically active components were identified (Figure 5A), of which 83 plant bioactive compounds were detected simultaneously in TCMSP and SymMap databases (Supplementary Table 2), and this cross-database overlap supports the reliability of predicted compounds. 237 intersecting therapeutic targets were obtained by Venn diagram analysis (Figure 5B). Gene Ontology (GO) enrichment analysis revealed that these targets were mainly involved in biological processes including lipid metabolism, inflammation and cell apoptosis, localized in organelles such as mitochondria and endoplasmic reticulum, and mediate molecular functions including kinase binding and inflammatory cytokine activation (Figure 5C). KEGG pathway enrichment analysis verified that the apoptosis pathway, lipid metabolism pathway, insulin resistance pathway and TNF inflammatory pathway are the core regulatory pathways (Figure 5D).

**FIGURE 5:**
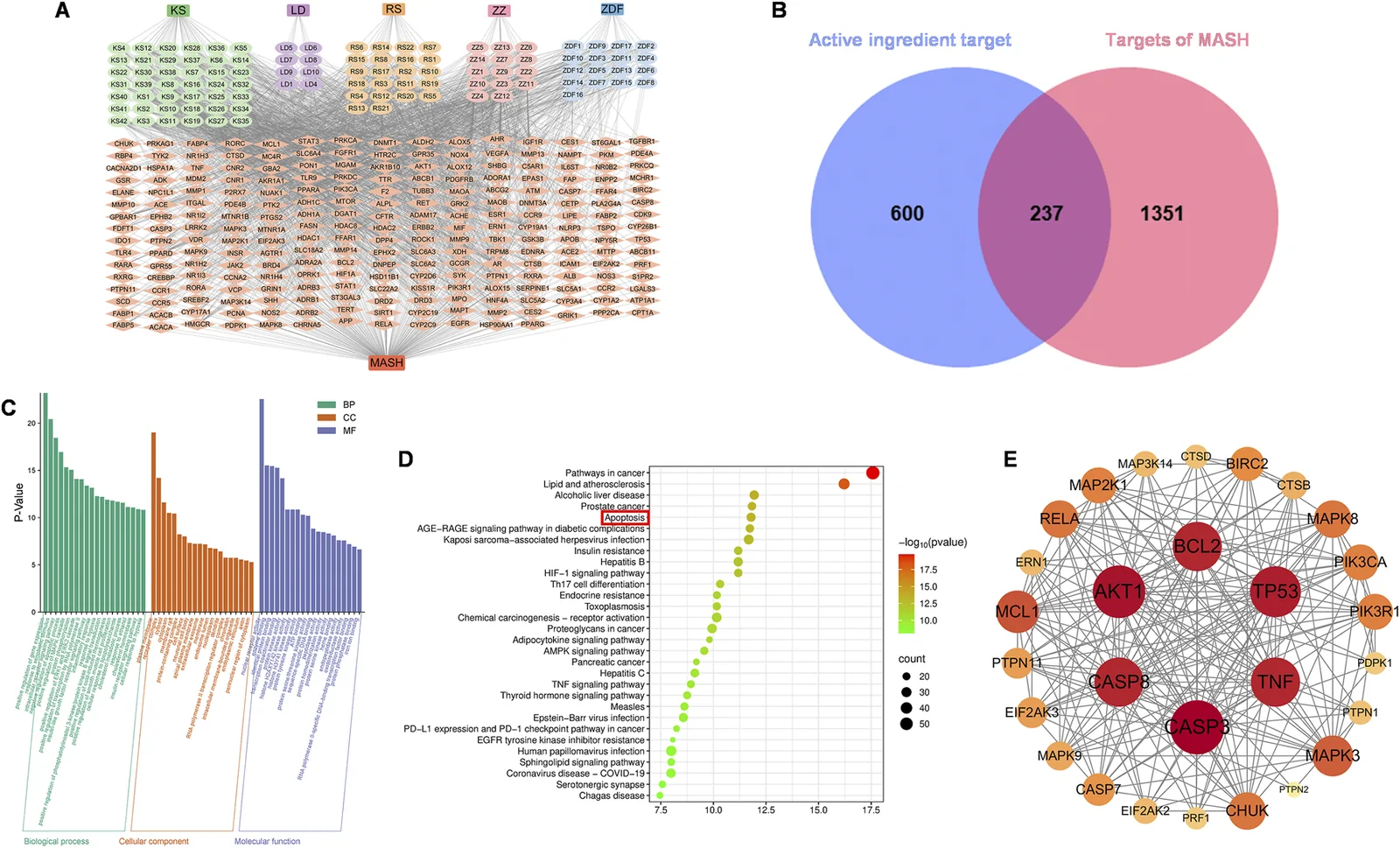
Apoptotic pathway of GDW against MASH elucidated by network pharmacology. **(A)** The herb-active ingredient-intersecting target-MASH disease interaction network is constructed. **(B)** The intersection of targets between GDW and MASH is identified. **(C)** The bar chart of GO functional enrichment (BP/CC/MF) for intersecting targets is presented. **(D)** The bubble plot of KEGG pathway enrichment for intersecting targets is shown. **(E)** The protein-protein interaction (PPI) network of core intersecting targets is established. KS, Sophorae Flavescentis Radix; LD, Gentianae Radix et Rhizoma; RS, Ginseng Radix et Rhizoma; ZZ, Gardeniae Fructus; ZDF, Suis Fellis Pulvis.

In summary, the intervention effect of GDW on MASH was achieved through the synergistic multiple target regulation of pathways related to apoptosis, inflammation, and lipid metabolism, among which the regulation of apoptosis was considered the core potential mechanism. Furthermore, it was found that within the apoptosis pathway, 19 key disease targets were modulated by GDW. Subsequent screening via protein-protein interaction (PPI) network analysis ultimately identified Caspase-3, AKT serine/threonine kinase 1 (AKT1), Tumor protein p53 (TP53), Caspase-8, Bcl-2, and TNF as core hub targets through which the progression of the disease was alleviated (Figure 5E).

### 3.6 GDW ameliorates MASH by modulating autophagy and attenuating hepatocyte apoptosis

Network pharmacology analysis identified apoptosis as a core pathway targeted by GDW in MASH. Given that autophagic dysfunction is recognized as an upstream driver of hepatocyte apoptosis (Cai et al., 2014; Tanaka et al., 2016; Wirawan et al., 2010), we examined the hepatic expression of autophagy markers (LC3 and p62) and key apoptotic proteins (Bcl-2, Caspase-3, and Caspase-8).

As shown in Figures 6A–C, LC3 expression was significantly elevated and p62 showed an upward trend in the model group, indicating impaired autophagic degradation and autophagy-related dysfunction. Following GDW treatment, the high-dose GDW group exhibited significantly reduced LC3 levels, and the low-dose GDW group showed decreased p62 expression, suggesting that GDW ameliorated this autophagy-related dysfunction.

**FIGURE 6:**
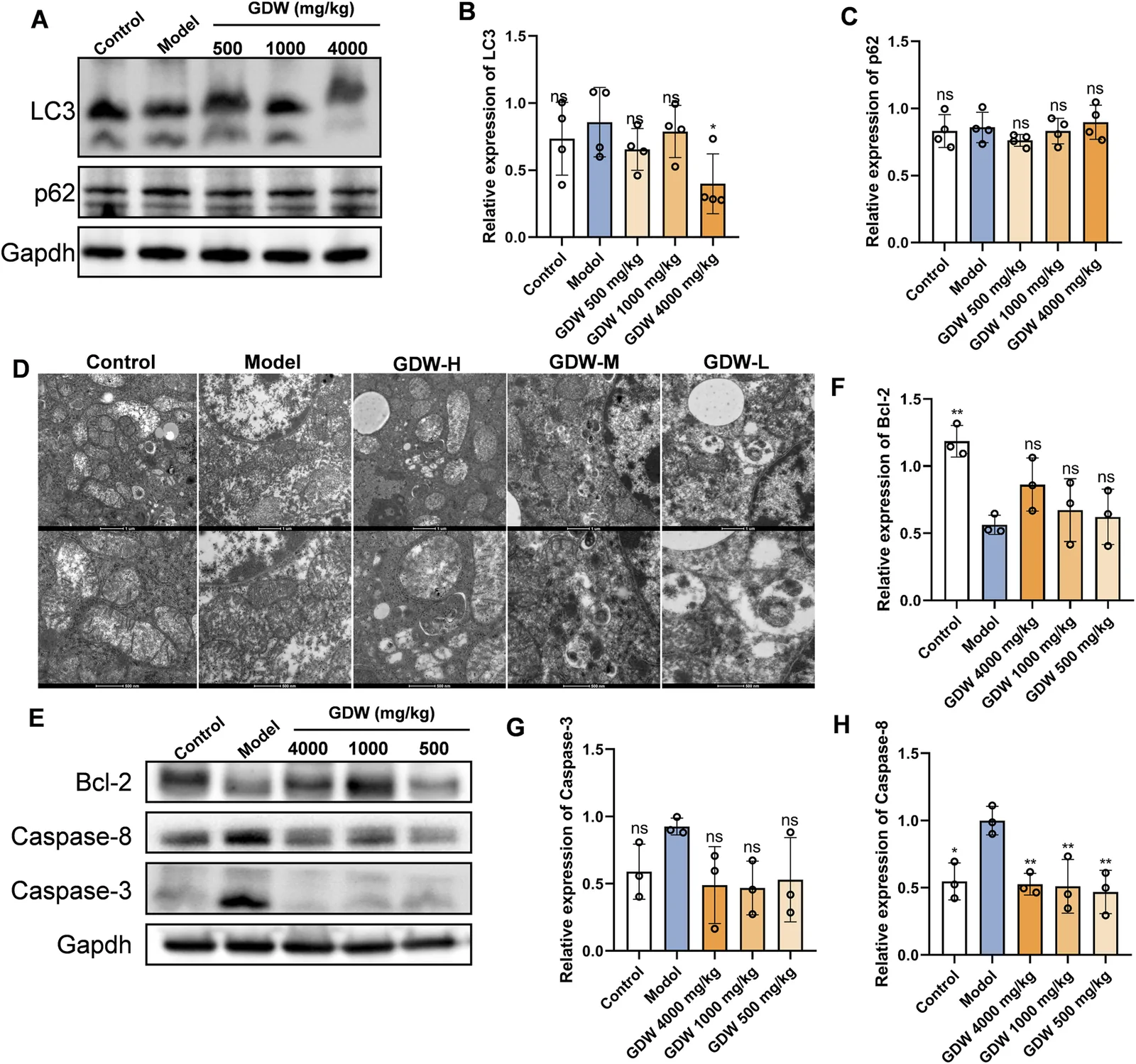
GDW ameliorates MASH by restoring autophagy and attenuating hepatocyte apoptosis. **(A–C, E–H)** The protein expression levels of LC3, p62, Bcl-2, Caspase-3 and Caspase-8 in the liver tissues of each group were detected by western blot analysis. **(D)** Representative TEM images of liver sections, scale bar, 1 μm (up), 500 nm (bottom). Control, normal control group; Model, MASH model group; Resmetirom, positive drug control group; GDW-H, M, L, GDW at 4000, 1000, and 500 mg/kg, respectively ns, no statistically significant difference, * *P* < 0.05 and **P < 0.01 vs. the model group.

TEM further provided ultrastructural evidence. In the model group, numerous damaged mitochondria with swelling and cristae rupture were observed, accompanied by reduced autolysosomes and accumulated undegraded mitochondria-containing autophagosomes. Following GDW intervention, damaged mitochondria were markedly decreased, autolysosome numbers were increased, and mitochondria engulfed and degraded by autolysosomes were visible (Figure 6D).

In parallel, we assessed apoptotic protein expression. Bcl-2 expression was significantly decreased in the model group, while the expressions of Caspase-3 and Caspase-8 were increased. GDW intervention significantly upregulated Bcl-2 and inhibited Caspase-3/8 expression, thereby blocking the activation of hepatocyte apoptosis (Figures 6E–H).

Collectively, GDW may modulate autophagic flux and enhance mitophagic activity to eliminate damaged mitochondria and reduce pro-apoptotic stimuli, thereby suppressing excessive hepatocyte apoptosis and ultimately ameliorating MASH.

### 3.7 Elucidation of the binding interactions between active ingredients of GDW and core targets based on UHPLC-QTOF-MS and molecular docking

UHPLC-QTOF-MS was employed for the systematic qualitative analysis of the overall chemical constituents of GDW. Based on accurate mass-to-charge ratios, tandem mass spectrometric fragmentation characteristics, and relevant literature data, a total of 179 chemical components (Supplementary Table 1) were identified, which were derived from the five constituent herbal medicines, namely, SFR, GERR, GF, GIRR, and SFP, and encompassed multiple structural types including iridoid glycosides, flavonoid glycosides, alkaloids, triterpenoid saponins, conjugated bile acids, and organic acids, thereby comprehensively reflecting the chemical diversity of GDW. Further hepatic distribution analysis indicated that 19 compounds were detected in liver (Table 1).

**TABLE 1:** Chemical components derived from GDW aqueous extract detected in hepatic tissues of MASH mice.

NO	T_R_ (min)	[M + H]^+^ (m/z)	ESI-MS^n^(+) (m/z)	[M-H]^-^(m/z)	ESI-MS^n^(−) (m/z)	Formula predicted	Predicted (m/z)	Measured (m/z)	Diff (ppm)	Assigned identification	Source	Liver
1	1.277	365.1054	-	​	​	C_12_H_22_O_11_	365.1054	365.1054	0.00	Gentiobiose	GERR	+
2	1.290	247.1807	179.1543, 245.1649, 150.1278, 148.1120	​	​	C_15_H_22_N_2_O	247.1805	247.1807	0.81	Sophocarpine	SFR	+
3	1.926	261.1599	114.0915	​	​	C_15_H_20_N_2_O_2_	261.1598	261.1599	0.38	Baptifoline	SFR	+
4	2.408	265.1912	247.0391, 205.1337, 136.0614	​	​	C_15_H_24_N_2_O_2_	265.1911	265.1912	0.38	5,6-Dehydrolupanine	SFR	+
5	3.264	245.1648	122.0958, 148.1122, 245.1648	​	​	C_15_H_20_N_2_O	245.1648	245.1648	0.00	Anagyrine	SFR	+
6	4.537	281.1861	245.1647, 148.1121, 263.1852	​	​	C_15_H_24_N_2_O_3_	281.186	281.1853	−2.49	Sophoranol N-oxide	SFR	+
7	4.600	249.1962	247.1903, 148.1119, 136.1122	​	​	C_15_H_24_N_2_O	249.1961	249.1962	0.40	Lupanine	SFR	+
8	5.823	249.1964	148.1115, 150.1278, 249.1964, 122.0961	​	​	C_15_H_24_N_2_O	249.1961	249.1964	1.20	Matrine	SFR	+
9	6.078	263.1755	136.1125, 150.1278, 245.1648, 148.1124	​	​	C_15_H_22_N_2_O_2_	263.1754	263.1755	0.38	Oxysophocarpine	SFR	+
10	6.778	265.1914	148.1119, 150.1276, 122.0965, 162.1279, 134.0957	​	​	C_15_H_24_N_2_O_2_	265.1911	265.1913	0.75	Oxymatrine/17-hydroxysophoridine	SFR	+
11	7.161	265.1911	247.1804, 205.1337, 150.1276, 136.1118, 148.1117	​	​	C_15_H_24_N_2_O_2_	265.1911	265.1913	0.75	Sophoranol/Hydroxylupanine	SFR	+
12	9.235	195.0653	177.0545, 149.0597, 121.0648	​	​	C_10_H_10_O_4_	195.0652	195.0653	0.51	Gentiopicral	GERR	+
13	1.127	​	​	377.0857	179.0559	C_18_H_17_O_9_	377.0867	377.0857	−2.65	Methylcorymbiferin/1-hydroxy-2,3,4,7-tetramethoxy xanthone	GERR	+
14	2.141	​	​	191.0195	111.9466	C_6_H_8_O_7_	191.0197	191.0195	−1.05	Citric acid	GERR	+
15	7.608	​	​	405.1418	179.0356	C_17_H_26_O_11_	405.1402	405.1418	3.95	Shanzhiside methyl ester	GERR	+
16	18.800	​	​	498.2893	498.2665	C_26_H_45_NO_6_S	498.2895	498.2893	−0.40	Taurohyodeoxycholic acid/Tauroursodeoxycholic acid	SFP	+
17	21.940	​	​	514.2858	496.2719	C_26_H_45_NO_7_S	514.2844	514.2858	2.72	Taurohyocholic acid	SFP	+
18	22.799	​	​	407.2796	325.1815	C_24_H_40_O_5_	407.2803	407.2796	−1.72	Cholic acid	SFP	+
19	35.993	​	​	437.1966	119.036	C_26_H_30_O_6_	437.197	437.1966	−0.91	Sophoraflavanone G	SFR	+

Among the 19 liver-distributed constituents, six compounds with a peak-area proportion >5% and well-defined chemical structures (anagyrine, lupanine, matrine, oxysophocarpine, sophocarpine, and taurohyocholic acid) were selected for molecular docking with the core targets of apoptosis pathways, namely, Caspase-3, AKT1, TP53, Caspase-8, Bcl-2, and TNF-α. A binding energy of ≤ −5 kcal/mol was set as the threshold for favorable ligand–receptor binding activity (Figure 7; Table 2). The results demonstrated that the binding energies of six representative core active components with Caspase-3, Tp53, AKT1 and TNF-α, ranged from -5.911 to -8.361 kcal/mol, all of which were below the threshold (−5.00 kcal/mol), suggesting favorable theoretical binding potential between these compounds and the corresponding targets.

**FIGURE 7:**
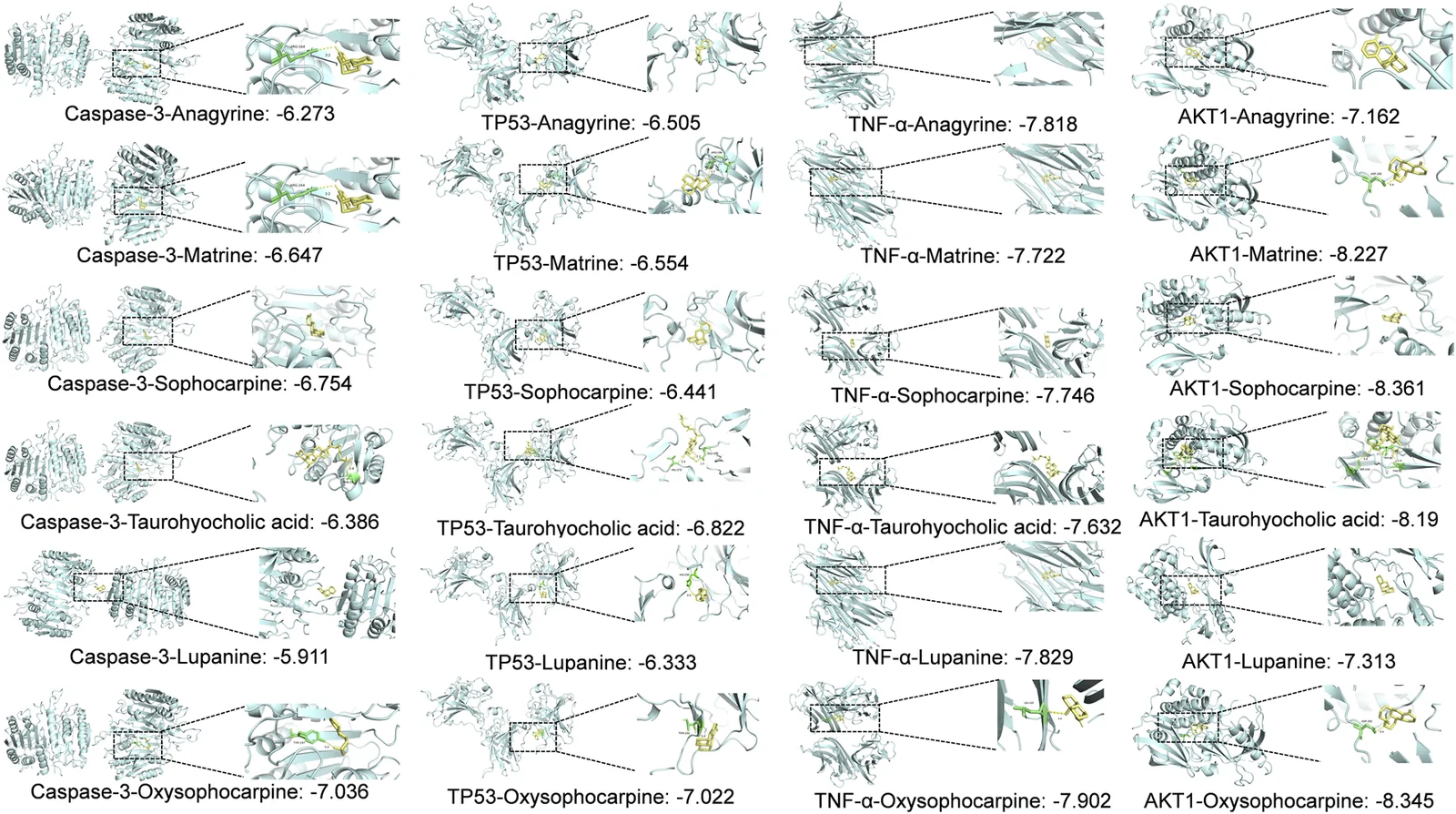
Binding modes obtained from molecular docking between core active constituents of GDW and key target proteins.

**TABLE 2:** Molecular docking results of core active constituents of GDW with key target proteins.

Target name	Ligand name	Docking score (kcal/mol)
Caspase-3	Anagyrine	−6.273
Caspase-3	Matrine	−6.647
Caspase-3	Oxysophocarpine	−7.036
Caspase-3	Sophocarpine	−6.754
Caspase-3	Taurohyocholic acid	−6.386
Caspase-3	Lupanine	−5.911
TNF-α	Anagyrine	−7.818
TNF-α	Matrine	−7.722
TNF-α	Oxysophocarpine	−7.902
TNF-α	Sophocarpine	−7.746
TNF-α	Taurohyocholic acid	−7.632
TNF-α	Lupanine	−7.829
AKT1	Anagyrine	−7.162
AKT1	Matrine	−8.227
AKT1	Oxysophocarpine	−8.345
AKT1	Sophocarpine	−8.361
AKT1	Taurohyocholic acid	−8.19
AKT1	Lupanine	−7.313
Bcl-2	Anagyrine	−4.526
Bcl2	Matrine	−4.407
Bcl2	Oxysophocarpine	−4.52
Bcl2	Sophocarpine	−4.365
Bcl2	Taurohyocholic acid	−4.949
Bcl2	Lupanine	−4.322
Caspase-8	Anagyrine	−4.489
Caspase-8	Matrine	−5.345
Caspase-8	Oxysophocarpine	−6.310
Caspase-8	Sophocarpine	−4.435
Caspase-8	Taurohyocholic acid	−4.839
Caspase-8	Lupanine	−5.293
TP53	Anagyrine	−6.505
TP53	Matrine	−6.554
TP53	Oxysophocarpine	−7.022
TP53	Sophocarpine	−6.441
TP53	Taurohyocholic acid	−6.822
TP53	Lupanine	−6.333

In summary, the chemical compositional features of GDW were systematically elucidated in this study by UHPLC-QTOF-MS, anagyrine, lupanine, matrine, oxysophocarpine, sophocarpine, and taurohyocholic acid were identified as the core pharmacologically effective substances that enter the liver. Furthermore, the molecular docking results further verified that the core active components could form stable bindings with key apoptotic and inflammatory regulatory targets, such as Caspase-3, AKT1, TP53 and TNF-α, through non-covalent interactions including hydrogen bonds and hydrophobic interactions, thereby preliminarily revealing the molecular mechanism underlying the “multi-component, multi-target” synergistic pharmacological effects of GDW, and providing a theoretical support for subsequent pharmacodynamic evaluation and in-depth mechanistic studies.

### 3.8 Effects of active ingredients from GDW on FFA-induced MASH in L02 cells

To screen the core bioactive monomers of GDW against MASH, FFA-induced MASH model was established in L02 hepatocytes. The regulatory effects of six candidate compounds including anagyrine, lupanine, matrine, oxysophocarpine, sophocarpine, and taurohyocholic acid, on hepatocyte injury, lipid accumulation, and inflammatory activation were systematically evaluated (Figure 8). The results showed that FFA induction markedly increased intracellular ALT and AST activities, elevated the levels of TC, TG, and LDL-C, and upregulated the expression of pro-inflammatory cytokines (TNF-α, IL-1β, and IL-6), indicating successful establishment of hepatocyte steatosis and inflammatory injury. After intervention with the compounds, matrine and taurohyocholic acid conferred comprehensive protection, reducing ALT/AST levels, ameliorating the dysregulation of TC, TG, LDL-C, and HDL-C, and markedly suppressing inflammatory cytokine expression, with combined anti-steatotic and anti-inflammatory activities. Sophocarpine and oxysophocarpine alleviated FFA-induced hepatocellular injury and inflammation, but showed limited lipid-regulating capacity, with no significant effects on HDL-C and only slight improvements in intracellular lipid accumulation. Anagyrine exerted strong hepatoprotective and anti-inflammatory effects, downregulating ALT/AST and multiple pro-inflammatory cytokines; however, it failed to regulate TG and only partially improved abnormalities in TC, LDL-C, and HDL-C. Lupanine displayed the weakest activity, only mildly ameliorating hepatocellular injury and some inflammatory indicators, with no significant effects on lipid parameters.

**FIGURE 8:**
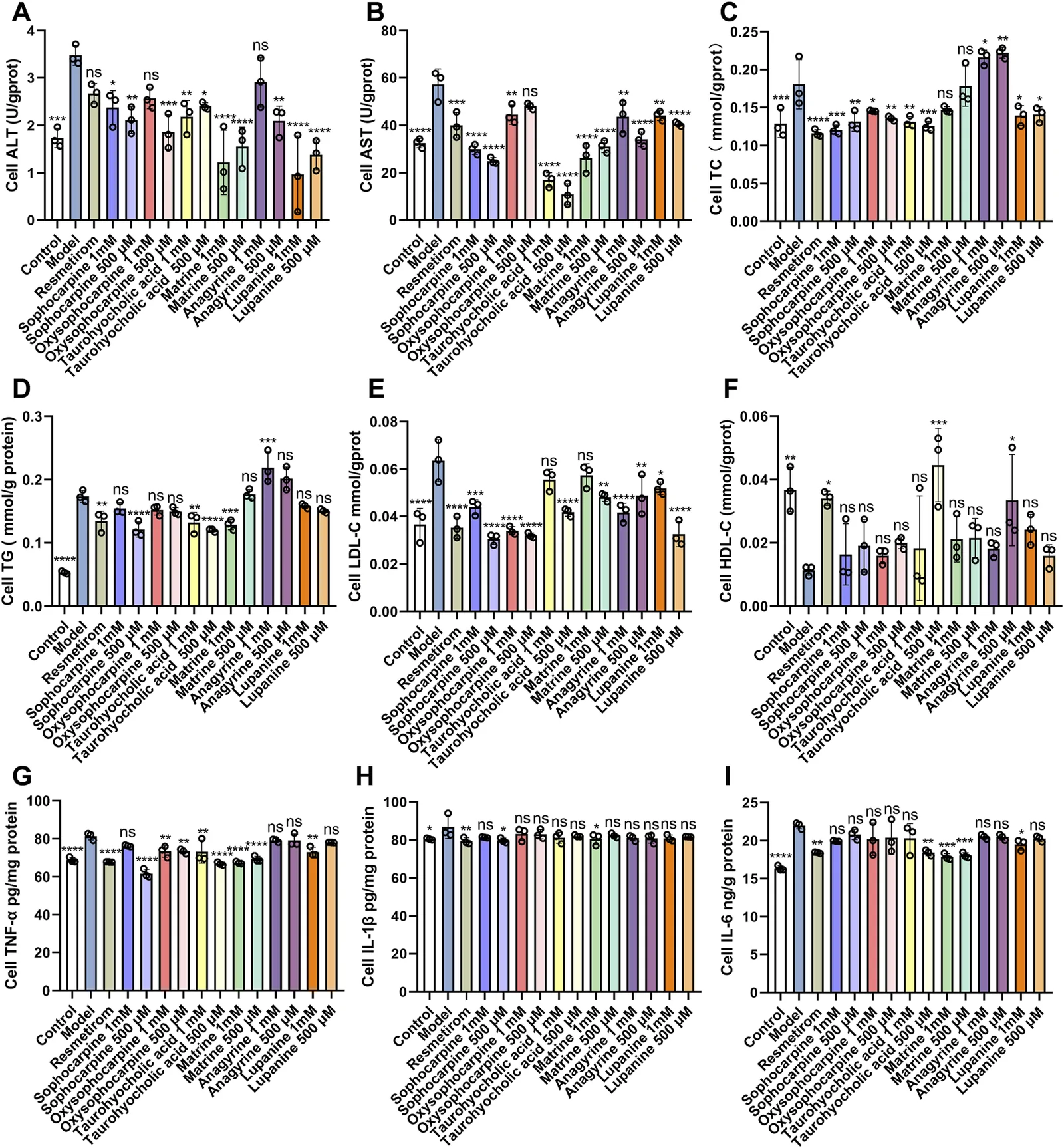
Effects of the main active ingredients from Gudan Wan on FFA-induced MASH in L02 hepatocytes. **(A,B)** Hepatocellular activities of ALT and AST. **(C–F)** Intracellular lipid levels of TC, TG, LDL-C, and HDL-C. **(G–I)** Pro-inflammatory cytokine levels of TNF-α, IL-1β, and IL-6. ns, no significant difference, * *P* < 0.05, ** *P* < 0.01, *** *P* < 0.001 and **** *P* < 0.0001 vs. the model group.

Collectively, these compounds may serve as candidate bioactive constituents through which GDW exerts its anti-MASH effects. These findings provide an experimental foundation for further mechanistic investigations and druggability assessments.

## 4 Discussion

MASH, a progressive form of MASLD, is characterized by aberrant hepatic lipid accumulation, accompanied by chronic inflammatory responses, hepatocellular injury, and variable degrees of fibrosis, and has emerged as one of the most prevalent chronic liver diseases worldwide (Devasia et al., 2025; Pipitone et al., 2023). Despite the rising global prevalence of MASH, specific therapeutic options remain scarce. Resmetirom and semaglutide have been approved for MASH intervention, their clinical response rates remain low, and their use is limited by gastrointestinal and cardiovascular risks (Harrison et al., 2024; Petta et al., 2025). TCM formulas, owing to their holistic regulatory advantages of multi-component, multi-target, and multi-pathway actions, have demonstrated unique therapeutic potential for MASH (Song et al., 2025). In this study, the intervention effects of GDW on MASH and its underlying molecular mechanisms were systematically evaluated through a 30-day toxicity test, pharmacodynamic evaluation in a MASH model, network pharmacology prediction, molecular biological validation, and molecular docking simulation.

Long-term toxic evaluation is recognized as a critical component of safety assessment in the research and development of new traditional Chinese medicines. In this study, a classic 30-day repeated-dose toxicity study design in rodents was adopted, and the *in vivo* safety of GDW at doses of 1000 mg/kg and 4000 mg/kg was systematically evaluated. No mortality or visible toxic reactions were observed in mice following 30 consecutive days of intragastric GDW administration. No obvious organic lesions were detected via histopathological examination of the heart, liver, spleen, lung, kidney, and brain tissues. No significant differences were identified in body weight, organ indices, and serum biochemical parameters (except BUN) between the test groups and the control group. Serum BUN levels in the mice of the treatment groups were significantly decreased, which may indicate that renal metabolism in mice is regulated to some extent by GDW, and that renal function may be improved or the kidney burden reduced (Xiang et al., 2026). The mechanistic and biological significance of this effect remains to be further investigated in subsequent studies. In summary, no evident organ toxicity was induced by long-term GDW administration within the tested dose range. The 30-day medication safety characteristics of GDW provide evidence of preclinical safety to support further investigation.

The HFD + CCl_4_-induced MASH mouse model, in which repeated CCl_4_ exposure serves as a second hit triggering oxidative stress, inflammation, and apoptosis and ultimately driving hepatic fibrosis, remains a widely used and reproducible tool in preclinical MASH research (Friedman et al., 2018). In this MASH mouse model, marked improvement in liver appearance was observed after GDW intervention, a downward trend was detected in liver index, and ALT and AST levels in both serum and liver were significantly reduced. Histopathological examination revealed that the severity of hepatocyte fatty vacuolar degeneration and inflammatory infiltration was alleviated. It was demonstrated by these results that MASH-induced liver injury can be effectively attenuated and hepatic tissue structure was restored by GDW. However, CCl_4_ is a direct hepatotoxin promoting hepatocyte injury and fibrosis. By introducing chemically induced injury, the HFD + CCl_4_ model cannot fully reproduce the metabolic features of human MASH, including chronic obesity and systemic insulin resistance. Further studies using MASH models induced by a high-fat diet alone or by other metabolic disturbances are therefore warranted to evaluate the therapeutic efficacy of GDW.

In view of the hepatoprotective effect of GDW on MASH, the effects of GDW on lipid metabolism, inflammation, and fibrosis in MASH mice were investigated. It was confirmed by the results that lipid metabolism disorders, inflammation, and fibrosis in MASH model mice were significantly reversed by GDW. Dysregulation of lipid metabolism leads to excess free fatty acid delivery to the liver, which drives mitochondrial dysfunction, endoplasmic reticulum stress, and lipid peroxidation, all of which converge to trigger hepatocyte injury and apoptotic cell death (Friedman et al., 2018). The pathological initiation of MASH is blocked at the source by GDW via correction of hepatic lipid homeostasis. Inflammatory response is regarded as the core driving force for the progression of MASH to fibrosis. The release of pro-inflammatory cytokines IL-6, TNF-α, and IL-1β in serum and liver tissues was significantly inhibited, hepatic inflammatory infiltration is reduced, and the inflammatory cascade amplification pathway is blocked by GDW. Hepatic stellate cells, activated by cytokines released from persistent inflammation, induce pathological deposition of type I and type III collagen, which is identified as a key node in the progression of MASH to liver cirrhosis (Wiering et al., 2023). As shown by Masson staining, the area of intrahepatic collagen deposition was significantly reduced and the degree of fibrosis was alleviated after GDW intervention. It is confirmed by the above *in vivo* pharmacodynamic results that, different from chemical single-target drugs, multiple pathological pathways including lipid metabolism, liver injury, inflammation, and fibrosis can be simultaneously regulated by GDW, which is consistent with the advantageous feature of traditional Chinese medicine formulas in the synergistic treatment of metabolic liver diseases with multiple components and multiple pathways.

Network pharmacology, as a systematic prediction tool, has been recognized as an effective strategy for elucidating the synergistic regulatory characteristics of multi-component, multi-target, and multi-pathway traditional Chinese medicine formulas (Hopkins, 2008). Through network pharmacology analysis, it was verified that the apoptosis pathway is one of the core pathways by which MASH is regulated by GDW. Hepatocyte apoptosis is considered a key pathological link in the progression of MASH from simple steatosis to inflammation and fibrosis. After the initiation of apoptosis, inflammatory response and hepatic stellate cell activation can be triggered by the release of apoptotic bodies, by which the inflammatory and fibrotic processes of MASH are further aggravated (Cheng et al., 2024). Caspase-3, AKT1, TP53, Caspase-8, Bcl-2, and TNF were finally identified as core targets via PPI network analysis. Among numerous molecules regulating apoptosis, the Bcl-2 protein family is identified as one of the core determinants of mitochondrial-mediated apoptosis. The apoptosis of mitochondrial pathway is regulated by this family through the precise balance between anti-apoptotic members (e.g., Bcl-2 and Bcl-xL) and pro-apoptotic members (e.g., BAX and BAK). It has been confirmed by existing studies that in animal models of fatty liver disease, the protein and mRNA expression levels of pro-apoptotic Bax are significantly upregulated in mouse liver tissues. Meanwhile, the level of Caspase-3 is increased as detected by immunohistochemistry, while the expression of anti-apoptotic Bcl-2 is inhibited. The abnormal expression levels of Bcl-2, Bax and Caspase-3 can be restored by the intervention of Fanlian Huazhuo Formula (Niu et al., 2024). Hepatocyte-specific knockout of Caspase-8 in mice is demonstrated to significantly alleviate hepatocellular apoptosis, inflammatory factor release and hepatic inflammatory infiltration (Hatting et al., 2013). Considering the core driving role of hepatocellular apoptosis in the pathological process of MASH, the direct execution and regulatory proteins of the apoptosis pathway, namely, Caspase-3, Caspase-8, and Bcl-2, were selected for *in vivo* experimental validation in this study. The results showed that the expression of the anti-apoptotic protein Bcl-2 was significantly decreased in the liver tissues of the model group, while the expressions of the pro-apoptotic execution proteins Caspase-3 and Caspase-8 were significantly increased. After GDW intervention, Bcl-2 expression was dose-dependently upregulated, and the aberrant activation of Caspase-3 and Caspase-8 was inhibited, thereby effectively blocking the initiation and execution of hepatocyte apoptosis.

MASH-related liver injury involves the coordinated participation of multiple modes of cell death, and apoptosis does not occur independently. Notably, impaired autophagy has been recognized as an early upstream pathogenic event in MASH, which disrupts lipid droplet clearance and leads to the accumulation of damaged mitochondria, thereby driving hepatocyte apoptosis (Akazawa and Nakao, 2018; Tanaka et al., 2016). Previous studies have found that in a methionine-choline-deficient diet induced MASH mouse model, blocked autophagic flux, as evidenced by p62 accumulation and an elevated LC3-II/LC3-I ratio, is accompanied by increased TUNEL-positive hepatocyte apoptosis (González-Rodríguez et al., 2014). Further interventional experiments at the cellular level confirmed that the autophagy inhibitor chloroquine or Atg5 silencing significantly aggravated Caspase-3 activation and apoptosis, whereas autophagy activation by rapamycin had the opposite effect (Cai et al., 2014), firmly demonstrating that autophagic dysfunction is a critical driver of MASH-related apoptotic injury. As canonical markers for evaluating autophagic flux, LC3 and p62/SQSTM1 are frequently found to be abnormally accumulated in MASH liver tissues (Klionsky et al., 2021). Such aberrant accumulation reflects impaired hepatic mitophagy, which exacerbates lipid deposition, oxidative stress, hepatocellular injury and apoptosis (Akazawa and Nakao, 2018; Carotti et al., 2020; González-Rodríguez et al., 2014). Consistent with these findings, our study showed that GDW treatment downregulated LC3 expression and reduced p62 levels, and TEM confirmed enhanced mitophagic clearance of damaged mitochondria. These results indicate that GDW ameliorates MASH-associated autophagy-related dysfunction, thereby suppressing excessive hepatocyte apoptosis and reducing intracellular lipid deposition (Figure 9). Although our findings suggest that GDW alleviates MASH by improving autophagic dysfunction, future studies using autophagic flux assays, such as the mCherry-GFP-LC3 dual-fluorescence reporter system, will provide growing evidence as to whether GDW exerts its therapeutic effects through the regulation of autophagic flux.

**FIGURE 9:**
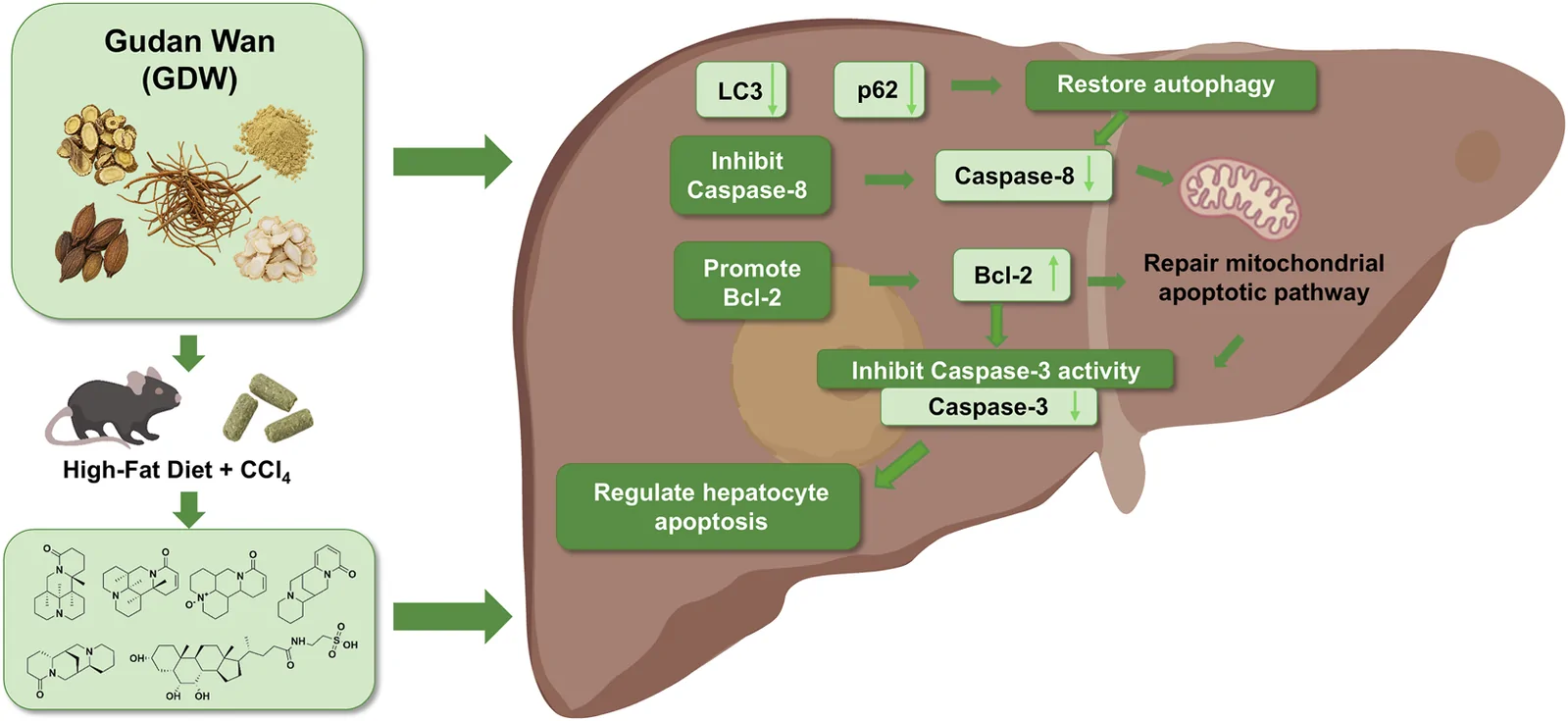
Schematic diagram of the proposed mechanism by which GDW attenuates MASH. GDW restores autophagy (as indicated by downregulation of LC3 and p62), promotes Bcl-2 expression, and inhibits Caspase-3/8 activation, thereby repairing mitochondrial dysfunction and suppressing hepatocyte apoptosis.

Furthermore, targets such as AKT1, TP53, and TNF serve as predictive findings at the network pharmacology level, providing directional reference for the potential multi-target regulatory mechanisms of GDW. These predictions are supported by previous experimental evidence. The Jianpi-Huayu-Jiedu Formula has been shown to ameliorate hepatic oxidative stress and lipid accumulation, thereby preventing MASH progression, via activation of the PI3K/AKT and PPAR pathways (Wu et al., 2025). Compound Dancao Granules have been shown to suppress the p53 signaling pathway, markedly ameliorate hepatic steatosis, inflammation and fibrosis in MASH mice, and inhibit hepatocellular apoptosis (Li et al., 2026). Additionally, TNF-α, as a key ligand of the death receptor pathway, participates in the inflammatory responses triggered by apoptotic bodies and is considered a pivotal driver of the inflammation-apoptosis loop in MASH (Cheng et al., 2024). Collectively, these results indicate that the therapeutic efficacy of GDW against MASH is at least partially attributable to the regulation of AKT1, TP53, and TNF-associated pathways, which provides a rationale for future mechanistic studies.

The therapeutic efficacy of traditional Chinese medicine formulas is achieved via the synergistic effects of multiple active components. Clarification of the interaction between the pharmacodynamic substances and core targets is a key step in elucidating the action basis of the formula. A total of 179 chemical components were identified from GDW by UHPLC-QTOF-MS (Supplementary Table 1), of which 19 were found to enter the liver. These components are derived from the five constituent herbal medicines of the formula, encompassing multiple structural types including iridoid glycosides, flavonoid glycosides, alkaloids, triterpenoid saponins, conjugated bile acids, and organic acids, which comprehensively reflect the chemical material basis of GDW. On this basis, molecular docking technology was further employed in this study to evaluate the theoretical binding capacity between the representative active components of GDW and the above-mentioned core targets predicted by network pharmacology. The results showed that the binding energies of the major active components in GDW, including anagyrine, lupanine, matrine, oxysophocarpine, sophocarpine, and taurohyocholic acid, to the four target proteins (Caspase-3, AKT1, TP53 and TNF-α) were determined to range from -5.911 to -8.361 kcal/mol, all below the threshold of −5.00 kcal/mol. Strong binding affinities between the core active components of GDW and the aforementioned proteins are indicated. For Bcl-2 and Caspase-8, the binding energies of most active components did not reach the threshold, as indicated by the molecular docking results, suggesting that the *in vivo* regulation of Bcl-2 and Caspase-8 by GDW might not be achieved through direct binding of active components, but more likely through indirect regulation via upstream signaling pathways. The specific regulatory mechanism remains to be further explored. Notably, high binding affinities between the major active substances of GDW and AKT1, TP53, and TNF were predicted by molecular docking, suggesting that these proteins might be potential targets of GDW active components. These compound-target interactions merit further evaluation using isothermal titration calorimetry, surface plasmon resonance, and cellular thermal shift assay. In addition, the anti-MASH effects of anagyrine, lupanine, matrine, oxysophocarpine, sophocarpine, and taurohyocholic acid have been confirmed by *in vitro* experiments, which further corroborates that the above active components are the pharmacodynamic material basis for GDW to exert anti-MASH effects. The remaining thirteen liver-distributed constituents might also contribute to the therapeutic effects of GDW against MASH. Among them, oxymatrine, gentian oligosaccharide, taurohyodeoxycholic acid, shanzhiside methyl ester, taurochenodeoxycholic acid, and citric acid have been reported to ameliorate MASH-related pathological features, including hepatic lipid accumulation, dysregulated lipid metabolism, oxidative stress, inflammation, fibrosis, impaired glucose homeostasis, and insulin resistance (Cui et al., 2023; Ghule, Kotagale and Patil, 2020; Li et al., 2025b; Lou et al., 2024; Yadikar et al., 2022; Zheng et al., 2024), while the roles of the other seven constituents in alleviating MASH-related pathological features have not yet been reported and deserve further investigation.

In summary, our results demonstrate for the first time that GDW alleviates MASH through the regulation of autophagy and the Bcl-2/caspase-3/caspase-8 apoptotic axis, despite the widely recognized fact that autophagic dysfunction drives hepatocyte apoptosis in the pathogenesis of MASH.

## 5 Conclusion

GDW demonstrated favorable *in vivo* safety following 30 days of repeated administration. GDW ameliorated hepatic function impairment, lipid metabolism disorders, and hepatic inflammation and fibrosis progression in MASH mice through the regulation of the apoptosis and autophagy. A total of 19 liver-directed components were identified in this study, among which the core constituents (such as anagyrine, lupanine, matrine, oxysophocarpine, sophocarpine, and taurohyocholic acid) exhibited relatively strong theoretical binding affinities with core targets of apoptosis, and exerted protective effects against free fatty acid-induced injury in L02 cells. Future investigations should be focused on the precise elucidation of active components in GDW, in-depth validation of key signaling pathways, and promotion of clinical translation and application, so as to provide safer and more effective traditional Chinese medicine regimens for the clinical treatment of MASH.

## Data Availability

The original contributions presented in the study are included in the article/Supplementary Material, further inquiries can be directed to the corresponding authors.

## Glossary

AKT1: Akt serine/threonine kinase 1
ALP: Alkaline phosphatase
ALT: Alanine aminotransferase
ANOVA: One-way analysis of variance
AST: Aspartate aminotransferase
BCA: Bicinchoninic acid assay
Bcl-2: B Cell lymphoma 2
BSA: Bovine serum albumin
BUN: Blood urea nitrogen
CCl_4_: Carbon tetrachloride
CREA: Creatinine
DMEM: Dulbecco’s modified eagle medium
ECL: Enhanced chemiluminescence
ESI: Electrospray ionization
FFA: Free fatty acids
GDW: Gudan wan
GERR: Gentianae radix et rhizoma
GF: Gardeniae fructus
GIRR: Ginseng radix et rhizoma
GLU: Glucose
GO: Gene ontology
H&E: Hematoxylin and eosin
HDL-C: High-density lipoprotein cholesterol
HFD: High-fat diet
HRP: Horseradish peroxidase
IL-1β: Interleukin-1 beta
IL-6: Interleukin-6
KEGG: Kyoto encyclopedia of genes and genomes
LC3: Microtubule-associated protein 1 light chain 3
LDL-C: Low-density lipoprotein cholesterol
LPS: Lipopolysaccharides
m/z: Mass-to-charge ratio
MASH: Metabolic dysfunction-associated steatohepatitis
MASLD: Metabolic dysfunction-associated steatotic liver disease
MS: Mass spectrometry
MS/MS: Tandem mass spectrometry
NF-κB: Nuclear factor kappa b
PBS: Phosphate-buffered saline
PI3K: Phosphoinositide 3-kinase
PPARα: Peroxisome proliferator-activated receptor α
PPI: Protein-protein interaction
PVDF: Polyvinylidene fluoride
RIPA: Radioimmunoprecipitation assay buffer
ROS: Reactive oxygen species
S.D.: Standard deviation
SDS-PAGE: Sodium dodecyl sulfate–polyacrylamide gel electrophoresis
SFP: Suis fellis pulvis
SFR: Sophorae flavescentis radix
SQSTM1 (p62): Sequestosome 1
TBST: Tris-buffered saline with tween-20
TC: Total cholesterol
TEM: Transmission electron microscope
TCM: Traditional Chinese medicine
TG: Triglycerides
TNF-α: Tumor necrosis factor α
TP53: Tumor protein p53
UHPLC-QTOF-MS: Ultra-high-performance liquid chromatography coupled with quadrupole time-of-flight mass spectrometry
