# Supplementary material for: Gudan Wan attenuates metabolic dysfunction-associated steatohepatitis by modulating autophagy and the Bcl-2/Caspase-3/Caspase-8 apoptotic axis
Source: Front Cell Dev Biol. 2026 Oct 22;14:1993264. doi: 10.3389/fcell.2026.1993264 (PMC13736602; doi:10.3389/fcell.2026.1993264)
Supplement: Supplementary file 1 [file Table1.docx]

**SUPPLEMENTARY MATERIAL**

**Gudan Wan Attenuates Metabolic Dysfunction-Associated Steatohepatitis by Modulating Autophagy and the Bcl-2/Caspase-3/Caspase-8 Apoptotic Axis**

Shaocong Han ^1, 2, 3, †^, Qiuyi Li^2, 3, †^, Zhongneng Ding^2, 3, †^, Huan Yang^2, 3^, Di Du^2, 3^, Qidi Zhou^2, 3^, Jie Yu ^1, 2, 3,^**^*^**, Xingxin Yang ^1, 2, 3,^**^*^**

^1^ The First Clinical College, Yunnan University of Chinese Medicine, 1076 Yuhua Road, Kunming 650500, China

^2^ College of Pharmaceutical Science, Yunnan University of Chinese Medicine, 1076 Yuhua Road, Kunming 650500, China

^3^ School of Chinese Materia Medica and Yunnan Key Laboratory of Southern Medicine Utilization, Yunnan University of Chinese Medicine, 1076 Yuhua Road, Kunming, 650500, China

^*^ Correspondence: yujie@ynucm.edu.cn (J. Y.); yxx78945@163.com (X.X.Y.)

^†^ These authors contributed equally to this work.

**Table of contents**

**Supplementary Experimental Procedures**

**Supplementary Figures and Tables**

1. **Table S1.** Chemical components of GDW aqueous extract detected in the herbal formula, as well as in the hepatic tissues and Hepatic mitochondria of MASH mice.
2. **Table S2.** Cross-validation of the active compounds of GDW identified from TCMSP using the SymMap database.

**Supplementary Experimental Procedures**

**Sample Preparation**

Liver tissue samples from the normal control group and the GDW-treated group were thawed at 4°C. Approximately 0.2 g of liver tissue from each individual sample was transferred into a homogenization tube and finely minced using sterile scissors. Subsequently, 1.0 mL of physiological saline was added, and the tissue was homogenized in an ice bath. The homogenate was transferred to a 5-mL centrifuge tube, mixed with 3.0 mL of acetonitrile, and vortexed for 30 s. The mixture was then incubated at 4°C for 20 min to facilitate protein precipitation.

All samples were centrifuged at 12,000 rpm for 15 min at 4°C. The supernatants were carefully collected into clean centrifuge tubes and evaporated to dryness under a gentle stream of nitrogen. The dried extracts from all individual samples within the same experimental group were combined into a single centrifuge tube and reconstituted with 200 μL of methanol. After vortex-mixing for 5 s, the solution was ultrasonicated for 20 min and centrifuged at 12,000 rpm for 10 min at 4°C. The resulting supernatant was collected for subsequent UPLC analysis.

**HPLC Conditions**

Chromatographic analysis was performed using an ACQUITY UPLC^®^ HSS T3 column (100 mm × 2.1 mm, 1.8 μm). The mobile phase consisted of acetonitrile (solvent A) and 0.1% formic acid in water (solvent B). Gradient elution was carried out as follows: 0-3 min, 3% A; 3-8 min, 3-20% A; 8-23 min, 20-45% A; 23-33 min, 45-65% A; 33-35 min, 65-100% A; and 35-40 min, 100% A. The flow rate was maintained at 0.2 mL/min, with an injection volume of 5 μL. The column temperature was set at 35°C, and detection was performed using a diode-array detector (DAD) at a wavelength of 200 nm.

**Table S1. Chemical components of GDW aqueous extract detected in the herbal formula, as well as in the hepatic tissues and Hepatic mitochondria of MASH mice.**

| **NO** | **T_R_**  **(min)** | **[M+H]^+^**  **(m/z)** | **ESI-MSn(+)**  **(m/z)** | **[M-H]^-^**  **(m/z)** | **ESI-MSn(-)**  **(m/z)** | **Formula Predicted** | **Predicted**  **(m/z)** | **Measured**  **(m/z)** | **Diff**  **(ppm)** | **Assigned identification** | **Source** | **Liver** |
| --- | --- | --- | --- | --- | --- | --- | --- | --- | --- | --- | --- | --- |
| 1 | 1.099 | 258.1101 | 104.1069 |  |  | C_22_H_26_O_8_ | 258.1101 | 258.1101 | 0.00 | Glycerophosphocholine | Gentianae Radix et Rhizoma | - |
| 2 | 1.277 | 365.1054 | - |  |  | C_12_H_22_O_11_ | 365.1054 | 365.1054 | 0.00 | Gentiobiose | Gentianae Radix et Rhizoma | + |
| 3 | 7.161 | 443.0946 | 281.1849, 263.1753, 247.1804 |  |  | C_20_H_20_O_10_Na | 443.0949 | 443.0946 | -0.68 | 8-O-glucosyl bellidifolin | Gentianae Radix et Rhizoma | - |
| 4 | 9.200 | 177.05455 | 149.0597, 131.049, 103.0541 |  |  | C_10_H_8_O_3_ | 177.0546 | 177.0545 | -0.56 | Erythrocentaurin | Gentianae Radix et Rhizoma | - |
| 5 | 9.235 | 195.0653 | 177.0545, 149.0597, 121.0648 |  |  | C_10_ H_10_ O_4_ | 195.0652 | 195.0653 | 0.51 | Gentiopicral | Gentianae Radix et Rhizoma | + |
| 6 | 9.328 | 586.1732 | 245.1642 |  |  | C_25_H_32_O_15_N | 586.1766 | 586.1732 | -5.80 | Gentiakochianoside | Gentianae Radix et Rhizoma | - |
| 7 | 11.557 | 493.1336 | 475.3266 |  |  | C_23_H_24_O_12_ | 493.1341 | 493.1336 | -1.01 | 6-C-B-D-glucospranosyltricine (isopyrenine) | Gentianae Radix et Rhizoma | - |
| 8 | 0.997 |  |  | 173.1041 | 131.0462 | C_6_H_14_N_4_O_2_ | 173.1044 | 173.1041 | -1.73 | Arginine | Gentianae Radix et Rhizoma | - |
| 9 | 1.066 |  |  | 697.1962 | 475.045 | C_31_H_38_O_18_ | 697.1985 | 697.1962 | -3.30 | Gentistraminoside A | Gentianae Radix et Rhizoma | - |
| 10 | 1.100 |  |  | 195.0508 | 129.0186 | C_6_H_12_O_7_ | 195.051 | 195.0508 | -1.03 | Gluconic acid | Gentianae Radix et Rhizoma | - |
| 11 | 1.127 |  |  | 377.0857 | 179.0559 | C_18_H_17_O_9_ | 377.0867 | 377.0857 | -2.65 | Methylcorymbiferin/ 1-hydroxy-2,3,4,7-tetramethoxy xanthone | Gentianae Radix et Rhizoma | + |
| 12 | 1.328 |  |  | 609.1830 | 179.0559, 343.1123, 503.1611 | C_28_H_34_O_15_ | 609.1825 | 609.183 | 0.82 | Hesperidin | Gentianae Radix et Rhizoma | - |
| 13 | 1.389 |  |  | 341.1089 | 179.0556, 161.0456 | C_12_H_22_O_11_ | 341.1089 | 341.1089 | 0.00 | Isomaltose | Gentianae Radix et Rhizoma | - |
| 14 | 1.455 |  |  | 503.1616 | 341.109, 179.0567 | C_18_H_32_O_16_ | 503.1618 | 503.1616 | -0.40 | Raffinose | Gentianae Radix et Rhizoma | - |
| 15 | 1.724 |  |  | 191.0194 | 111.0085 | C_6_H_8_O_7_ | 191.0197 | 191.0194 | -1.57 | Citric acid | Gentianae Radix et Rhizoma | - |
| 16 | 2.092 |  |  | 503.1615 | 341.1084 | C_18_H_32_O_16_ | 503.1618 | 503.1615 | -0.60 | Gentianose | Gentianae Radix et Rhizoma | - |
| 17 | 5.619 |  |  | 373.1142 | 193.0494 | C_16_H_22_O_10_ | 373.114 | 373.1142 | 0.54 | Geniposidic acid | Gentianae Radix et Rhizoma | - |
| 18 | 5.951 |  |  | 375.1284 | 151 | C_16_H_23_O_10_ | 375.1286 | 375.1284 | -0.53 | Loganic acid | Gentianae Radix et Rhizoma | - |
| 19 | 6.192 |  |  | 193.0508 | 149.0610 | C_10_H_10_O_4_ | 193.0506 | 193.0508 | 1.04 | Ferulic acid/ Isoferulic acid | Gentianae Radix et Rhizoma | - |
| 20 | 6.321 |  |  | 179.0348 | 135.0453 | C_9_H_8_O_4_ | 179.0350 | 179.0348 | -1.12 | Caffeic acid | Gentianae Radix et Rhizoma | - |
| 21 | 6.795 |  |  | 315.0722 | 153.0205 | C_13_H_16_O_9_ | 315.0722 | 315.0722 | 0.00 | Vanilloloside/ Gentisic acid-5-O-β-glucoside or  5-(β-D-glucopyranosyl)-2-hydroxybenzoic acid | Gentianae Radix et Rhizoma | - |
| 22 | 6.855 |  |  | 405.1426 | 179.0424 | C_17_H_26_O_11_ | 405.1402 | 405.1426 | 5.92 | Caryptoside | Gentianae Radix et Rhizoma | - |
| 23 | 7.246 |  |  | 403.1232 | 339.0055 | C_17_H_23_O_11_ | 403.1235 | 403.1232 | -0.74 | Sweroside/ Gardenoside | Gentianae Radix et Rhizoma | - |
| 24 | 7.608 |  |  | 405.1418 | 179.0356 | C_17_H_26_O_11_ | 405.1402 | 405.1418 | 3.95 | Shanzhiside methyl ester | Gentianae Radix et Rhizoma | + |
| 25 | 7.639 |  |  | 537.1829 | 179.0353, 375.1306 | C_22_H_34_O_15_ | 537.1825 | 537.1829 | 0.74 | Gmephiloside | Gentianae Radix et Rhizoma | - |
| 26 | 7.790 |  |  | 375.1295 | 213.0761 | C_16_H_24_O_10_ | 375.1297 | 375.1295 | -0.53 | Mussaenosidic acid | Gentianae Radix et Rhizoma | - |
| 27 | 7.820 |  |  | 537.1841 | 268.0683 | C_22_H_34_O_15_ | 537.1825 | 537.1841 | 2.98 | Strychnicacid-11-O-β-glucopyranosyl ester | Gentianae Radix et Rhizoma | - |
| 28 | 8.031 |  |  | 537.1823 | 151.0392, 213.0759 | C_22_H_34_O_15_ | 537.1825 | 537.1823 | -0.37 | Loganic Acid 6'-Glucoside | Gentianae Radix et Rhizoma | - |
| 29 | 8.182 |  |  | 491.1391 | 459.0865 | C_20_H_28_O_14_ | 491.1406 | 491.1391 | -3.05 | 2'-o-(2,3-dihyrben)-gentiopicroside | Gentianae Radix et Rhizoma | - |
| 30 | 8.242 |  |  | 537.1809 | 213.075 | C_22_H_34_O_15_ | 537.1825 | 537.1809 | -2.98 | 6-glu-Loganic acid | Gentianae Radix et Rhizoma | - |
| 31 | 8.303 |  |  | 389.1083 | 165.0551, 345.1508 | C_16_H_22_O_11_ | 389.1089 | 389.1083 | -1.54 | Secologanosideor Cleft ring-loganin/ Cleft ring-loganin | Gentianae Radix et Rhizoma | - |
| 32 | 8.484 |  |  | 563.1607 | 193.0516, 165.0546 | C_23_H_31_O_16_ | 563.1607 | 563.1607 | 0.00 | 6'-O-β-D-glucosyl gentiopicroside | Gentianae Radix et Rhizoma | - |
| 33 | 8.755 |  |  | 419.1185 | 149.06, 179.036, 193.0518 | C_17_H_23_O_12_ | 419.1184 | 419.1185 | 0.24 | Swertiamarin | Gentianae Radix et Rhizoma | - |
| 34 | 9.057 |  |  | 609.1455 | 429.0972, 447.1071, 519.1702 | C_27_H_29_O_16_ | 609.1450 | 609.1455 | 0.82 | Isoorientin-4’-O-β-D-glucosyle/ 4'-glu-isoorientin | Gentianae Radix et Rhizoma | - |
| 35 | 9.087 |  |  | 387.1294 | 193.0496 | C_17_H_24_O_10_ | 387.1297 | 387.1294 | -0.77 | 3-EpiswertiajaposideC | Gentianae Radix et Rhizoma | - |
| 36 | 9.117 |  |  | 401.1078 | 149.061, 165.0188 | C_17_H_21_O_11_ | 401.1078 | 401.1078 | 0.00 | Gentiopicroside | Gentianae Radix et Rhizoma | - |
| 37 | 9.147 |  |  | 609.1443 | 447.1063 | C_27_H_30_O_16_ | 609.1461 | 609.1443 | -2.96 | Rutin/ Lutonarin | Gentianae Radix et Rhizoma | - |
| 38 | 9.178 |  |  | 403.1126 | 388.1324 | C_24_H_20_O_6_ | 403.1187 | 403.1126 | -15.13 | Tribenzoin | Gentianae Radix et Rhizoma | - |
| 39 | 9.298 |  |  | 401.1089 | 179.0564 | C_17_H_22_O_11_ | 401.1089 | 401.1089 | 0.00 | Ixoside | Gentianae Radix et Rhizoma | - |
| 40 | 9.419 |  |  | 403.1238 | 179.0354 | C_17_H_24_O_11_ | 403.1236 | 403.1238 | 0.50 | 6-β-hydroxyswertiajaposide A/ 8-epikingside or  Gentiascabraside A | Gentianae Radix et Rhizoma | - |
| 41 | 9.570 |  |  | 593.1500 | 503.0978 | C_27_H_29_O_15_ | 593.1501 | 593.15 | -0.17 | Saponarin | Gentianae Radix et Rhizoma | - |
| 42 | 9.570 |  |  | 593.1500 | 503.0978 | C_27_H_30_O_15_ | 593.1512 | 593.15 | -2.02 | Isosaponarin | Gentianae Radix et Rhizoma | - |
| 43 | 9.600 |  |  | 655.1864 | 475.1052 | C_29_H_35_O_17_ | 655.1869 | 655.1864 | -0.76 | Gentiotrifloroside | Gentianae Radix et Rhizoma | - |
| 44 | 9.610 |  |  | 593.1511 | 503.0956 | C_27_H_30_O_15_ | 593.1512 | 593.1511 | -0.17 | Vicenin-2/ Isovitexin-6''-O-glucoside | Gentianae Radix et Rhizoma | - |
| 45 | 9.691 |  |  | 595.1648 | 475.104 | C_27_H_32_O_15_ | 595.1668 | 595.1648 | -3.36 | Naringenin-7-O-gentiobioside | Gentianae Radix et Rhizoma | - |
| 46 | 10.143 |  |  | 581.2246 | 401.1095 | C_28_H_38_O_13_ | 581.224 | 581.2246 | 1.03 | Tortoside B | Gentianae Radix et Rhizoma | - |
| 47 | 10.234 |  |  | 739.2097 | 315.0304, 577.1541613.1318 | C_33_H_40_O_19_ | 739.2091 | 739.2097 | 0.81 | 3-deacetyltrifloroside | Gentianae Radix et Rhizoma | - |
| 48 | 10.566 |  |  | 623.1610 | 503.1415 | C_28_H_32_O_16_ | 623.1618 | 623.161 | -1.28 | Isoscoparin-2''-O-glucopyranoside | Gentianae Radix et Rhizoma | - |
| 49 | 10.776 |  |  | 269.0453 | 119.0362 | C_21_H_26_O_6_ | 269.0455 | 269.0453 | -0.74 | Apigenin | Gentianae Radix et Rhizoma | - |
| 50 | 10.837 |  |  | 581.1506 | 401.1091 | C_26_H_30_O_15_ | 581.1512 | 581.1506 | -1.03 | Gentianabavaroside | Gentianae Radix et Rhizoma | - |
| 51 | 10.927 |  |  | 551.1414 | 491.1184 | C_25_H_28_O_14_ | 551.1406 | 551.1414 | 1.45 | Dideacetylate-deglu-rindoside | Gentianae Radix et Rhizoma | - |
| 52 | 10.987 |  |  | 431.0978 | 255.0487, 283.0602, 387.0497 | C_21_H_20_O_10_ | 431.0984 | 431.0978 | -1.39 | Isoviexin | Gentianae Radix et Rhizoma | - |
| 53 | 11.018 |  |  | 581.1512 | 401.1129 | C_26_H_30_O_15_ | 581.1512 | 581.1512 | 0.00 | Gentiabavaroside | Gentianae Radix et Rhizoma | - |
| 54 | 11.048 |  |  | 521.2041 | 178.9977 | C_26_H_34_O_11_ | 521.2028 | 521.2041 | 2.49 | Lariciresinol-4-O-glucoside | Gentianae Radix et Rhizoma | - |
| 55 | 11.168 |  |  | 581.1713 | 563.1415 | C_23_H_33_O_17_ | 581.1712 | 581.1713 | 0.17 | 6'-O-β-D-glucosyl swertiamarin | Gentianae Radix et Rhizoma | - |
| 56 | 11.199 |  |  | 269.0446 | 225.0765 | C_15_H_10_O_5_ | 269.0445 | 269.0446 | 0.37 | Aloe-emodin | Gentianae Radix et Rhizoma | - |
| 57 | 11.289 |  |  | 655.1879 | 609.148, 493.133, 315.0299 | C_29_H_36_O_17_ | 655.1880 | 655.1879 | -0.15 | 6’-O-[3-(β-Dglucopyranosyloxy)-2hydroxybenzoyl]sweroside | Gentianae Radix et Rhizoma | - |
| 58 | 11.440 |  |  | 449.1076 | 283.0602 | C_21_H_21_O_11_ | 449.1078 | 449.1076 | -0.45 | 2-C-β-D-glucospranosyl-glucopyranosyl -1-hydroxy-7-methoxyxanthone | Gentianae Radix et Rhizoma | - |
| 59 | 11.440 |  |  | 153.0192 | 109.0297 | C_7_H_6_O_4_ | 153.0193 | 153.0192 | -0.65 | Protocatechuic acid | Gentianae Radix et Rhizoma | - |
| 60 | 11.742 |  |  | 299.0568 | 176.9613, 191.9452 | C_16_H_12_O_6_ | 299.0561 | 299.0568 | 2.34 | Kaempferide/ Hispidulin | Gentianae Radix et Rhizoma | - |
| 61 | 12.013 |  |  | 187.0976 | 125.0974 | C_9_H_16_O_4_ | 187.0976 | 187.0976 | 0.00 | Azelaic acid | Gentianae Radix et Rhizoma | - |
| 62 | 12.043 |  |  | 417.1551 | 387.0494 | C_22_H_26_O_8_ | 417.1555 | 417.1551 | -0.96 | (+)-Syringaresinol | Gentianae Radix et Rhizoma | - |
| 63 | 12.345 |  |  | 431.0974 | 283.0622 | C_21_H_19_O_10_ | 431.0973 | 431.0974 | 0.23 | Isovitexin | Gentianae Radix et Rhizoma | - |
| 64 | 12.557 |  |  | 737.1927 | 575.1426 | C_33_H_38_O_19_ | 737.1935 | 737.1927 | -1.09 | 2-deaceyl-triflorside | Gentianae Radix et Rhizoma | - |
| 65 | 12.617 |  |  | 755.2018 | 153.0187 | C_33_H_40_O_20_ | 755.204 | 755.2018 | -2.91 | Gentistraminoside B | Gentianae Radix et Rhizoma | - |
| 66 | 13.328 |  |  | 285.0406 | 112.9856, 174.9563, 267.0656 | C_15_H_10_O_6_ | 285.0405 | 285.0406 | 0.35 | Kaempferol | Gentianae Radix et Rhizoma | - |
| 67 | 15.651 |  |  | 491.1195 | 153.0188 | C_23_H_24_O_12_ | 491.1195 | 491.1195 | 0.00 | Methyl-3-[(6-O-β-D-glucopyranosyl)-β-D-glucopyranosyloxy]-2-hydroxybenzoate | Gentianae Radix et Rhizoma | - |
| 68 | 15.712 |  |  | 535.1454 | 153.0187, 491.1186 | C_25_H_28_O_13_ | 535.1457 | 535.1454 | -0.56 | 6'-O-D-glu-swertiamarin | Gentianae Radix et Rhizoma | - |
| 69 | 18.106 |  |  | 797.2132 | 515.2879 | C_35_H_41_O_21_ | 797.2135 | 797.2132 | -0.38 | Rindoside | Gentianae Radix et Rhizoma | - |
| 70 | 18.951 |  |  | 781.2198 | 577.1549, 697.2660 | C_35_H_42_O_20_ | 781.2197 | 781.2198 | 0.13 | Trifloroside | Gentianae Radix et Rhizoma | - |
| 71 | 19.663 |  |  | 875.2244 | 577.1461 | C_40_H_44_O_22_ | 875.2251 | 875.2244 | -0.80 | Isomacrophylloside | Gentianae Radix et Rhizoma | - |
| 72 | 19.699 |  |  | 875.2241 | 577.1596 | C_40_H_43_O_22_ | 875.2241 | 875.2241 | 0.00 | Macrophylloside A | Gentianae Radix et Rhizoma | - |
| 73 | 19.866 |  |  | 499.3056 | 455.2027 | C_30_H_44_O_6_ | 499.3065 | 499.3056 | -1.80 | 11-Deoxocucurbitacin I | Gentianae Radix et Rhizoma | - |
| 74 | 24.088 |  |  | 391.2852 | 191.9467 | C_24_H_40_O_4_ | 391.2854 | 391.2852 | -0.51 | Deoxycholic acid | Gentianae Radix et Rhizoma | - |
| 75 | 24.399 |  |  | 635.1608 | 493.2807 | C_29_H_32_O_16_ | 635.1618 | 635.1608 | -1.57 | Deglucogelidoside | Gentianae Radix et Rhizoma | - |
| 76 | 19.663 |  |  | 875.2244 | 577.1461 | C_40_H_44_O_22_ | 875.2251 | 875.2244 | -0.80 | Isomacrophylloside | Gentianae Radix et Rhizoma | - |
| 77 | 14.203 |  |  | 945.5408 | 475.1216 | C_48_H_82_O_18_ | 945.5428 | 945.5408 | -2.12 | Ginsenoside Re | Ginseng Radix et Rhizoma | - |
| 78 | 19.633 |  |  | 1107.5935 | 783.2274 | C_54_H_92_O_23_ | 1107.5957 | 1107.5935 | -1.99 | Ginsenoside Rb1a | Ginseng Radix et Rhizoma | - |
| 79 | 20.354 |  |  | 955.49 | 455.2029 | C_48_H_76_O_19_ | 955.4908 | 955.49 | -0.84 | Ginsenoside Ro | Ginseng Radix et Rhizoma | - |
| 80 | 18.8 |  |  | 498.2893 | 498.2665 | C_26_H_45_NO_6_S | 498.2895 | 498.2893 | -0.40 | Taurohyodeoxycholic acid/ Tauroursodeoxycholic acid | Suis Fellis Pulvis | + |
| 81 | 20.587 |  |  | 464.3019 | 446.2895 | C_26_H_43_NO_6_ | 464.3018 | 464.3019 | 0.22 | Glycocholic acid | Suis Fellis Pulvis | - |
| 82 | 21.078 |  |  | 448.3068 | 402.0473 | C_26_H_43_NO_5_ | 448.3068 | 448.3068 | 0.00 | Glycohyodeoxycholic acid | Suis Fellis Pulvis |  |
| 83 | 21.52 |  |  | 448.3067 | 404.3167 | C_26_H_43_NO_5_ | 448.3068 | 448.3067 | -0.22 | Glycochenodeoxycholic Acid | Suis Fellis Pulvis |  |
| 84 | 21.94 |  |  | 514.2858 | 496.2719 | C_26_H_45_NO_7_S | 514.2844 | 514.2858 | 2.72 | Taurohyocholic acid | Suis Fellis Pulvis | + |
| 85 | 23.128 |  |  | 407.2802 | 389.0467, 353.1395 | C_24_H_40_O_5_ | 407.2803 | 407.2802 | -0.25 | Cholic acid/ Hyocholic acid | Suis Fellis Pulvis | - |
| 86 | 23.519 |  |  | 464.3019 | 446.3352 | C_26_H_43_NO_6_ | 464.3018 | 464.3019 | 0.22 | Glycocholic acid | Suis Fellis Pulvis | - |
| 87 | 27.045 |  |  | 391.2848 | 389.2691, 353.1019 | C_24_H_40_O_4_ | 391.2854 | 391.2848 | -1.53 | Chenodeoxycholic Acid | Suis Fellis Pulvis | - |
| 88 | 29.606 |  |  | 391.2847 | 325.1843 | C_24_H_40_O_4_ | 391.2854 | 391.2847 | -1.79 | Deoxycholic acid | Suis Fellis Pulvis | - |
| 89 | 1.611 |  |  | 659.1611 | 497.1113 | C_31_H_32_O_16_ | 659.1618 | 659.1611 | -1.06 | 3,4-Dicaffeoy 1-5-(3-hydrox y-3-methylglu taroyl) quinic acid | Gardeniae Fructus | - |
| 90 | 3.178 |  |  | 429.0821 | 208.9706 | C_21_H_18_O_10_ | 429.0827 | 429.0821 | -1.40 | Daidzein-7-O-glucuronide | Gardeniae Fructus | - |
| 91 | 6.312 |  |  | 361.1503 | 181.0491 | C_16_H_26_O_9_ | 361.1504 | 361.1503 | -0.28 | Jasminoside Fisomers | Gardeniae Fructus | - |
| 92 | 6.373 |  |  | 391.1244 | 121.0300, 149.0612, 193.0511 | C_16_H_24_O_11_ | 391.1246 | 391.1244 | -0.51 | Shanzhiside | Gardeniae Fructus | - |
| 93 | 7.307 |  |  | 345.1555 | 165.0924 | C_16_H_26_O_8_ | 345.1555 | 345.1555 | 0.00 | JasminosideD | Gardeniae Fructus | - |
| 94 | 7.397 |  |  | 403.125 | 241.0725 | C_17_H_24_O_11_ | 403.1246 | 403.125 | 0.99 | Deacetyl asperulosidic acid methyl ester | Gardeniae Fructus | - |
| 95 | 7.427 |  |  | 345.1552 | 165.0919 | C_16_H_26_O_8_ | 345.1555 | 345.1552 | -0.87 | jasminoside A/E | Gardeniae Fructus | - |
| 96 | 7.457 |  |  | 403.1251 | 241.0726 | C_17_H_24_O_11_ | 403.1246 | 403.1251 | 1.24 | Gardenoside | Gardeniae Fructus | - |
| 97 | 7.488 |  |  | 345.1554 | 165.0547 | C_16_H_26_O_8_ | 345.1555 | 345.1554 | -0.29 | Picrocrocinic acid | Gardeniae Fructus | - |
| 98 | 7.669 |  |  | 405.1377 | 139.0762, 193.0514 | C_17_H_24_O_11_ | 405.1391 | 405.1377 | -3.46 | 7 ,8-epoxy-8a-dihydrogeniposide | Gardeniae Fructus | - |
| 99 | 7.759 |  |  | 375.1286 | 193.0502 | C_16_H_22_O_10_ | 375.1286 | 375.1286 | 0.00 | Gardoside | Gardeniae Fructus | - |
| 100 | 8.061 |  |  | 345.1555 | 151.0016, 165.0553 | C_16_H_26_O_8_ | 345.1555 | 345.1555 | 0.00 | JasminosideB | Gardeniae Fructus | - |
| 101 | 8.333 |  |  | 549.182 | 207.0657, 255.0583 | C_23_H_34_O_15_ | 549.1825 | 549.182 | -0.91 | Genipin gentiobioside | Gardeniae Fructus | - |
| 102 | 8.514 |  |  | 389.109 | 165.0554 | C_16_H_22_O_11_ | 389.1089 | 389.109 | 0.26 | Deacetyl-asperulosidic acid | Gardeniae Fructus | - |
| 103 | 8.815 |  |  | 551.1944 | 101.0241, 123.0446, 225.0765 | C_23_H_34_O_15_ | 551.197 | 551.1944 | -4.72 | Genipin-1-β-D-gentiobioside | Gardeniae Fructus | - |
| 104 | 9.087 |  |  | 387.1294 | 207.0760, 2225.0699 | C_17_H_24_O_10_ | 387.1297 | 387.1294 | -0.77 | Geniposide | Gardeniae Fructus | - |
| 105 | 9.268 |  |  | 225.0768 | 207.0664 | C_11_H_14_O_5_ | 225.0768 | 225.0768 | 0.00 | Genipin | Gardeniae Fructus | - |
| 106 | 9.298 |  |  | 401.1089 | 193.0508, 207.0662, 225.0766 | C_17_H_22_O_11_ | 401.1089 | 401.1089 | 0.00 | Genipin-1-O-glucuronide | Gardeniae Fructus | - |
| 107 | 9.57 |  |  | 403.1251 | 191.9476 | C_17_H_24_O_11_ | 403.1246 | 403.1251 | 1.24 | Scandoside methyl ester | Gardeniae Fructus | - |
| 108 | 9.751 |  |  | 519.1506 | 119.0363, 123.0449, 149.9681 | C_25_H_28_O_12_ | 519.1508 | 519.1506 | -0.39 | 6-O-trans-coumaroyl geniposidic acid | Gardeniae Fructus | - |
| 109 | 9.781 |  |  | 447.0927 | 119.0358 | C_21_H_20_O_11_ | 447.0933 | 447.0927 | -1.34 | Naringenin-7-O-glucuronide | Gardeniae Fructus | - |
| 110 | 9.872 |  |  | 447.092 | 119.0357 | C_21_H_20_O_11_ | 447.0933 | 447.092 | -2.91 | Naringenin-4-O-glucuronide | Gardeniae Fructus | - |
| 111 | 9.902 |  |  | 387.1293 | 101.0246, 123.0450, 207.0663 | C_17_H_24_O_10_ | 387.1297 | 387.1293 | -1.03 | 8-epiapodantheroside | Gardeniae Fructus | - |
| 112 | 10.897 |  |  | 183.1027 | 165.0552 | C_10_H_16_O_3_ | 183.1027 | 183.1027 | 0.00 | Jasminodiol | Gardeniae Fructus | - |
| 113 | 11.229 |  |  | 269.045 | 225.0749 | C_15_H_10_O_5_ | 269.0455 | 269.045 | -1.86 | Emodin | Gardeniae Fructus | - |
| 114 | 11.561 |  |  | 579.172 | 119.0368 | C_27_H_32_O_14_ | 579.1719 | 579.172 | 0.17 | Isonaringin | Gardeniae Fructus | - |
| 115 | 11.561 |  |  | 579.172 | 123.0447, 367.0997 | C_27_H_32_O_14_ | 579.172 | 579.172 | 0.00 | 6-O-trans-sinapoyl gardoside | Gardeniae Fructus | - |
| 116 | 11.681 |  |  | 579.1719 | 119.0356 | C_27_H_32_O_14_ | 579.1719 | 579.1719 | 0.00 | Naringin | Gardeniae Fructus | - |
| 117 | 13.147 |  |  | 975.3716 | 651.2607 | C_44_H_64_O_24_ | 975.3715 | 975.3716 | 0.10 | trans-crocin4/cis-crocin4 | Gardeniae Fructus | - |
| 118 | 13.147 |  |  | 975.3716 | 651.2607 | C_44_H_64_O_24_ | 975.3715 | 975.3716 | 0.10 | CrocinA | Gardeniae Fructus | - |
| 119 | 13.962 |  |  | 167.107 | 121.0289 | C_10_H_16_O_2_ | 167.1078 | 167.107 | -4.79 | crocusatin-C | Gardeniae Fructus | - |
| 120 | 15.018 |  |  | 253.0509 | 208.9342 | C_15_H_10_O_4_ | 253.0506 | 253.0509 | 1.19 | Daidzein | Gardeniae Fructus | - |
| 121 | 19.163 |  |  | 975.3715 | 651.2567, 697.2685 | C_44_H_64_O_24_ | 975.3715 | 975.3715 | 0.00 | Icrocin-Ⅰ | Gardeniae Fructus | - |
| 122 | 1.163 | 205.1337 | 104.1071 |  |  | C_12_H_16_N_2_O | 205.1335 | 205.1337 | 0.98 | N-methylcytisine | Sophorae Flavescentis Radix | - |
| 123 | 1.227 | 281.1860 | 263.1754, 136.0614 |  |  | C_15_H_24_N_2_O_3_ | 281.186 | 281.186 | 0.00 | Sophcence A | Sophorae Flavescentis Radix | - |
| 124 | 1.290 | 247.1807 | 179.1543, 245.1649, 150.1278, 148.1120 |  |  | C_15_H_22_N_2_O | 247.1805 | 247.1807 | 0.81 | Sophocarpine | Sophorae Flavescentis Radix | + |
| 125 | 1.608 | 281.1861 | 136.062, 245.1649, 263.1757, 203.0527 |  |  | C_15_H_24_N_2_O_3_ | 281.186 | 281.1861 | 0.36 | 5α,9α-dihydroxymatrine | Sophorae Flavescentis Radix | - |
| 126 | 1.862 | 191.1178 | 150.1278, 148.0759 |  |  | C_11_H_14_N_2_O | 191.1179 | 191.1178 | -0.52 | Cytisine | Sophorae Flavescentis Radix | - |
| 127 | 1.926 | 261.1599 | 114.0915 |  |  | C_15_H_20_N_2_O_2_ | 261.1598 | 261.1599 | 0.38 | Baptifoline | Sophorae Flavescentis Radix | + |
| 128 | 1.990 | 223.1444 | 191.1182, 114.0911 |  |  | C_12_H_18_N_2_O_2_ | 223.1441 | 223.1444 | 1.34 | Kuraramine/ Isokuraramine | Sophorae Flavescentis Radix | - |
| 129 | 2.408 | 265.1912 | 247.0391, 205.1337, 136.0614 |  |  | C_15_H_24_N_2_O_2_ | 265.1911 | 265.1912 | 0.38 | 5,6-Dehydrolupanine | Sophorae Flavescentis Radix | + |
| 130 | 2.499 | 281.1862 | 246.1688, 150.1277, 136.0617 |  |  | C_15_H_24_N_2_O_3_ | 281.186 | 281.1856 | -1.42 | 5-Hydroxycytisine | Sophorae Flavescentis Radix | - |
| 131 | 3.264 | 245.1648 | 122.0958, 148.1122, 245.1648, |  |  | C_15_H_20_N_2_O | 245.1648 | 245.1648 | 0.00 | Anagyrine | Sophorae Flavescentis Radix | + |
| 132 | 3.709 | 249.1961 | 249.2063, 136.1117, 148.1121, 247.1912 |  |  | C_15_H_24_N_2_O | 249.1961 | 249.1961 | 0.00 | Sophoridine | Sophorae Flavescentis Radix | - |
| 133 | 4.155 | 281.1862 | 263.1753, 150.1277, 136.112 |  |  | C_15_H_24_N_2_O_3_ | 281.186 | 281.1853 | -2.49 | 9-hydroxylamprolobine | Sophorae Flavescentis Radix | - |
| 134 | 4.155 | 249.1960 | 150.1277, 148.1117, 136.1120 |  |  | C_15_H_24_N_2_O | 249.1961 | 249.196 | -0.40 | Tetrahydroneosophoramine | Sophorae Flavescentis Radix | - |
| 135 | 4.537 | 281.1861 | 245.1647, 148.1121, 263.1852 |  |  | C_15_H_24_N_2_O_3_ | 281.186 | 281.1853 | -2.49 | Sophoranol N-oxide | Sophorae Flavescentis Radix | + |
| 136 | 4.6 | 249.1962 | 247.1903, 148.1119, 136.1122 |  |  | C_15_H_24_N_2_O | 249.1961 | 249.1962 | 0.40 | Lupanine | Sophorae Flavescentis Radix | + |
| 137 | 5.657 | 281.1862 | 263.1762, 150.1277, 136.1118 |  |  | C_15_H_24_N_2_O_3_ | 281.186 | 281.1868 | 2.85 | Epilamprolobine N-oxide | Sophorae Flavescentis Radix | - |
| 138 | 5.759 | 247.1808 | 148.1123 |  |  | C_15_H_22_N_2_O | 247.1805 | 247.1808 | 1.21 | 7,11-Dehydromatrine/ Lemannine | Sophorae Flavescentis Radix | - |
| 139 | 5.823 | 249.1964 | 148.1115, 150.1278, 249.1964, 122.0961 |  |  | C_15_H_24_N_2_O | 249.1961 | 249.1964 | 1.20 | Matrine | Sophorae Flavescentis Radix | + |
| 140 | 5.951 | 263.1755 | 245.1647 |  |  | C_15_H_22_N_2_O_2_ | 263.1754 | 263.1755 | 0.38 | Leontalbinine N-oxide/ Manmanine | Sophorae Flavescentis Radix | - |
| 141 | 6.078 | 263.1755 | 136.1125, 150.1278, 245.1648, 148.1124 |  |  | C_15_H_22_N_2_O_2_ | 263.1754 | 263.1755 | 0.38 | Oxysophocarpine | Sophorae Flavescentis Radix | + |
| 142 | 6.396 | 265.1913 | 150.1279, 247.1806 |  |  | C_15_H_24_N_2_O_2_ | 265.1911 | 265.1913 | 0.75 | 9α-Hydroxymatrine | Sophorae Flavescentis Radix | - |
| 143 | 6.396 | 263.1757 | 245.1651, 120.0805 |  |  | C_15_H_22_N_2_O_2_ | 263.1754 | 263.1757 | 1.14 | 9α-hydroxysophocarpine/ 5α-hydroxysophocarpine/ 12α-hydroxysophocarpine | Sophorae Flavescentis Radix | - |
| 144 | 6.778 | 265.1914 | 148.1119, 150.1276, 122.0965, 162.1279, 134.0957 |  |  | C_15_H_24_N_2_O_2_ | 265.1911 | 265.1913 | 0.75 | Oxymatrine/ 17-Hydroxysophoridine | Sophorae Flavescentis Radix | + |
| 145 | 7.033 | 247.1806 | 108.0805, 122.0966 |  |  | C_15_H_22_N_2_O | 247.1805 | 247.1806 | 0.40 | Isosophocarpine | Sophorae Flavescentis Radix | - |
| 146 | 7.161 | 265.1911 | 247.1804, 205.1337, 150.1276, 136.1118, 148.1117 |  |  | C_15_H_24_N_2_O_2_ | 265.1911 | 265.1913 | 0.75 | Sophoranol/ Hydroxylupanine | Sophorae Flavescentis Radix | + |
| 147 | 7.607 | 209.1651 | 105.0701, 122.0966 |  |  | C_12_H_20_N_2_O | 209.1648 | 209.1651 | 1.43 | flavascensine isomer | Sophorae Flavescentis Radix | - |
| 148 | 8.881 | 265.1911 | 150.1276, 263.1753 |  |  | C_15_H_24_N_2_O_2_ | 265.1911 | 265.1913 | 0.75 | Lamprolobine/ Oxysophoridine | Sophorae Flavescentis Radix | - |
| 149 | 9.838 | 261.1598 | 122.097, 120.0228, |  |  | C_15_H_20_N_2_O_2_ | 261.1598 | 261.1598 | 0.00 | 9α-hydroxysophoramine | Sophorae Flavescentis Radix | - |
| 150 | 12.703 | 283.0608 | 249.1958 |  |  | C_16_H_10_O_5_ | 283.0601 | 283.06 | -0.35 | pseudobaptigenin | Sophorae Flavescentis Radix | - |
| 151 | 14.044 | 255.065 |  |  |  | C_15_H_10_O_4_ | 255.0652 | 255.0654 | 0.78 | Daidzein | Sophorae Flavescentis Radix | - |
| 152 | 15.700 | 285.0756 | 137.0233 |  |  | C_16_H_12_O_5_ | 285.0757 | 285.076 | 1.05 | 7,4'-Dihydroxy-3'-methoxyisoflavone | Sophorae Flavescentis Radix | - |
| 153 | 10.264 |  |  | 283.0611 | 268.0368 | C_16_H_12_O_5_ | 283.0612 | 283.0611 | -0.35 | Stamens isoflavones | Sophorae Flavescentis Radix | - |
| 154 | 10.475 |  |  | 577.1561 | 283.0611 | C_27_H_30_O_14_ | 577.1562 | 577.1561 | -0.17 | 3'-hydroxy kushenol O | Sophorae Flavescentis Radix | - |
| 155 | 10.535 |  |  | 283.0613 | 268.0368, 283.0613 | C_16_H_12_O_5_ | 283.0612 | 283.0613 | 0.35 | Maackiain | Sophorae Flavescentis Radix | - |
| 156 | 10.746 |  |  | 283.0607 | 268.0368 | C_16_H_12_O_5_ | 283.0612 | 283.0607 | -1.77 | Calycosin | Sophorae Flavescentis Radix | - |
| 157 | 10.806 |  |  | 563.1407 | 269.0445 | C_26_H_28_O_14_ | 563.1406 | 563.1407 | 0.18 | 5-Hydroxy-2-(4hydroxyphenyl)-4-oxo-4Hchromen-7-yl 2-O[(2S,3S,4R)-3,4-dihydroxy-4(hydroxymethyl)tetrahydro-2furanyl]-β-D-glucopyranoside | Sophorae Flavescentis Radix | - |
| 158 | 11.108 |  |  | 563.14 | 269.0447, 255.0501 | C_26_H_28_O_14_ | 563.1406 | 563.14 | -1.07 | Apiin | Sophorae Flavescentis Radix | - |
| 159 | 11.440 |  |  | 153.0192 | 109.0297 | C_7_H_6_O_4_ | 153.0193 | 153.0192 | -0.65 | 3,4-Dihydroxybenzoic acid | Sophorae Flavescentis Radix | - |
| 160 | 12.864 |  |  | 267.0661 | 252.0426 | C_16_H_12_O_4_ | 267.0662 | 267.0661 | -0.37 | 7-methoxy-4'-hydroxyisoflavone | Sophorae Flavescentis Radix | - |
| 161 | 12.960 |  |  | 561.1611 | 267.0663, 252.0429 | C_27_H_30_O_13_ | 561.1613 | 561.1611 | -0.36 | 7-hydroxy-3′'-methoxyisoflavone-7-primeveroside | Sophorae Flavescentis Radix | - |
| 162 | 13.178 |  |  | 267.0658 | 252.0425 | C_16_H_12_O_4_ | 267.0663 | 267.0658 | -1.87 | Formononetin | Sophorae Flavescentis Radix | - |
| 163 | 13.359 |  |  | 561.1614 | 267.066, 252.0422 | C_27_H_30_O_13_ | 561.1613 | 561.1614 | 0.18 | Kushenol O | Sophorae Flavescentis Radix | - |
| 164 | 13.871 |  |  | 421.2036 | 271.0672 | C_26_H_30_O_5_ | 421.202 | 421.2036 | 3.80 | Kushenol R/ Kushenol U | Sophorae Flavescentis Radix | - |
| 165 | 14.233 |  |  | 271.0612 | 135.0059 | C_15_H_12_O_5_ | 271.0611 | 271.0612 | 0.37 | Naringenin | Sophorae Flavescentis Radix | - |
| 166 | 16.406 |  |  | 471.2015 | 135.0054 | C_26_H_32_O_8_ | 471.2024 | 471.2015 | -1.91 | Kushenol H/ Kushenol K | Sophorae Flavescentis Radix | - |
| 167 | 22.06 |  |  | 353.1399 | 119.0366 | C_21_H_22_O_5_ | 353.1394 | 353.1399 | 1.42 | Xanthohumol | Sophorae Flavescentis Radix | - |
| 168 | 24.129 |  |  | 453.1916 | 177.0189 | C_26_H_30_O_7_ | 453.1918 | 453.1916 | -0.44 | Kushenol I/ Kushenol N | Sophorae Flavescentis Radix | - |
| 169 | 25.777 |  |  | 439.1752 | 161.0241, 275.1653, | C_25_H_28_O_7_ | 439.1762 | 439.1752 | -2.28 | Kushenol L | Sophorae Flavescentis Radix | - |
| 170 | 26.028 |  |  | 437.1966 | 146.9628 | C_26_H_30_O_6_ | 437.197 | 437.1965 | -1.14 | Kuraridine/ Kurarinone | Sophorae Flavescentis Radix | - |
| 171 | 26.572 |  |  | 339.1231 | 191.9468, 133.0288 | C_20_H_20_O_5_ | 339.1238 | 339.1231 | -2.06 | Demethylxanthohumol/ Sophoraflavanone B | Sophorae Flavescentis Radix | - |
| 172 | 27.010 |  |  | 353.1035 | 161.0244 | C_20_H_18_O_6_ | 353.1031 | 353.1035 | 1.13 | Noranhydroicaritin | Sophorae Flavescentis Radix | - |
| 173 | 29.319 |  |  | 423.1815 | 261.1493 | C_25_H_28_O_6_ | 423.1813 | 423.1815 | 0.47 | Sophoraflavanone L | Sophorae Flavescentis Radix | - |
| 174 | 29.432 |  |  | 437.1604 | 176.9622 | C_25_H_26_O_7_ | 437.1606 | 437.1604 | -0.46 | Kushenol C | Sophorae Flavescentis Radix | - |
| 175 | 30.556 |  |  | 423.1809 | 161.0241 | C_25_H_28_O_6_ | 423.1813 | 423.1809 | -0.95 | Norkurarinone | Sophorae Flavescentis Radix | - |
| 176 | 33.55 |  |  | 423.1809 | 161.0242 | C_25_H_28_O_6_ | 423.1813 | 423.1809 | -0.95 | Sophoraflavanone G (Kushenol F) | Sophorae Flavescentis Radix | - |
| 177 | 33.654 |  |  | 437.1966 | 161.0239 | C_26_H_30_O_6_ | 437.197 | 437.1966 | -0.91 | Isokuraridine | Sophorae Flavescentis Radix | - |
| 178 | 34.890 |  |  | 507.2389 | 339.1974 | C_30_H_36_O_7_ | 507.2388 | 507.2389 | 0.20 | Kushenol M | Sophorae Flavescentis Radix | - |
| 179 | 35.749 |  |  | 423.1815 | 161.0237 | C_25_H_28_O_6_ | 423.1813 | 423.1815 | 0.47 | Kushenol E | Sophorae Flavescentis Radix | - |

**Table S2. Cross-validation of the active compounds of GDW identified from TCMSP using the SymMap database.**

| **NO.** | **Mol ID** | **Molecule Name** | **Source** | **SymMap** |
| --- | --- | --- | --- | --- |
| 1 | MOL001040 | (2R)-5,7-dihydroxy-2-(4-hydroxyphenyl)chroman-4-one | Sophorae Flavescentis Radix | + |
| 2 | MOL001484 | Inermine | Sophorae Flavescentis Radix | + |
| 3 | MOL003542 | 8-Isopentenyl-kaempferol | Sophorae Flavescentis Radix | + |
| 4 | MOL003627 | Sophocarpine | Sophorae Flavescentis Radix | + |
| 5 | MOL003648 | Inermin | Sophorae Flavescentis Radix | + |
| 6 | MOL003673 | Wighteone | Sophorae Flavescentis Radix | + |
| 7 | MOL003676 | Sophoramine | Sophorae Flavescentis Radix | + |
| 8 | MOL003680 | Sophoridine | Sophorae Flavescentis Radix | + |
| 9 | MOL000392 | Formononetin | Sophorae Flavescentis Radix | + |
| 10 | MOL004580 | Cis-Dihydroquercetin | Sophorae Flavescentis Radix | + |
| 11 | MOL004941 | (2R)-7-hydroxy-2-(4-hydroxyphenyl)chroman-4-one | Sophorae Flavescentis Radix | + |
| 12 | MOL005100 | 5,7-dihydroxy-2-(3-hydroxy-4-methoxyphenyl)chroman-4-one | Sophorae Flavescentis Radix | + |
| 13 | MOL005944 | Matrine | Sophorae Flavescentis Radix | + |
| 14 | MOL000006 | Luteolin | Sophorae Flavescentis Radix | + |
| 15 | MOL006561 | (+)-14α-hydroxymatrine | Sophorae Flavescentis Radix | + |
| 16 | MOL006562 | (+)-7,11-dehydromatrine,(leontalbinine) | Sophorae Flavescentis Radix | + |
| 17 | MOL006563 | (+)-9α-hydroxymatrine | Sophorae Flavescentis Radix | + |
| 18 | MOL006564 | (+)-allomatrine | Sophorae Flavescentis Radix | + |
| 19 | MOL006565 | AIDS211310 | Sophorae Flavescentis Radix | + |
| 20 | MOL006566 | (+)-lehmannine | Sophorae Flavescentis Radix | + |
| 21 | MOL006568 | Isosophocarpine | Sophorae Flavescentis Radix | + |
| 22 | MOL006569 | (-)-14β-hydroxymatrine | Sophorae Flavescentis Radix | + |
| 23 | MOL006570 | (-)-9α-hydroxysophoramine | Sophorae Flavescentis Radix | + |
| 24 | MOL006571 | Anagyrine | Sophorae Flavescentis Radix | + |
| 25 | MOL006572 | 1,4-diazaindan-type,alkaloid,flavascensine | Sophorae Flavescentis Radix | + |
| 26 | MOL006573 | 13,14-dehydrosophoridine | Sophorae Flavescentis Radix | + |
| 27 | MOL006582 | 5α,9α-dihydroxymatrine | Sophorae Flavescentis Radix | + |
| 28 | MOL006596 | Glyceollin | Sophorae Flavescentis Radix | + |
| 29 | MOL003347 | Hyperforin | Sophorae Flavescentis Radix | + |
| 30 | MOL006604 | (2S)-7-hydroxy-2-(4-hydroxyphenyl)-5-methoxy-8-(3-methylbut-2-enyl)chroman-4-one | Sophorae Flavescentis Radix | + |
| 31 | MOL006613 | Kushenin | Sophorae Flavescentis Radix | + |
| 32 | MOL006622 | Kushenol O | Sophorae Flavescentis Radix | + |
| 33 | MOL006623 | Kushenol,t | Sophorae Flavescentis Radix | + |
| 34 | MOL006626 | Leachianone,g | Sophorae Flavescentis Radix | + |
| 35 | MOL006627 | Lehmanine | Sophorae Flavescentis Radix | + |
| 36 | MOL006628 | (+)-Lupanine | Sophorae Flavescentis Radix | + |
| 37 | MOL006630 | Norartocarpetin | Sophorae Flavescentis Radix | + |
| 38 | MOL000456 | Phaseolin | Sophorae Flavescentis Radix | + |
| 39 | MOL006649 | Sophranol | Sophorae Flavescentis Radix | + |
| 40 | MOL006650 | (-)-Maackiain-3-O-glucosyl-6'-O-malonate | Sophorae Flavescentis Radix | + |
| 41 | MOL006652 | Trifolrhizin | Sophorae Flavescentis Radix | + |
| 42 | MOL000098 | Quercetin | Sophorae Flavescentis Radix | + |
| 43 | MOL001558 | Sesamin | Gentianae Radix et Rhizoma | + |
| 44 | MOL002322 | Isovitexin | Gentianae Radix et Rhizoma | + |
| 45 | MOL003137 | Leucanthoside | Gentianae Radix et Rhizoma | + |
| 46 | MOL003143 | Gentirigenic acid | Gentianae Radix et Rhizoma | + |
| 47 | MOL003152 | Gentisin | Gentianae Radix et Rhizoma | + |
| 48 | MOL003155 | Pranferin | Gentianae Radix et Rhizoma | + |
| 49 | MOL003169 | Gentiopicroside tetraacetate | Gentianae Radix et Rhizoma | + |
| 50 | MOL003170 | Gentisein | Gentianae Radix et Rhizoma | + |
| 51 | MOL000359 | Sitosterol | Gentianae Radix et Rhizoma | + |
| 52 | MOL000422 | Kaempferol | Gentianae Radix et Rhizoma | + |
| 53 | MOL002879 | Diop | Ginseng Radix et Rhizoma | + |
| 54 | MOL000449 | Stigmasterol | Ginseng Radix et Rhizoma | + |
| 55 | MOL004492 | Chrysanthemaxanthin | Ginseng Radix et Rhizoma | + |
| 56 | MOL005308 | Aposiopolamine | Ginseng Radix et Rhizoma | + |
| 57 | MOL005314 | Celabenzine | Ginseng Radix et Rhizoma | + |
| 58 | MOL005317 | Deoxyharringtonine | Ginseng Radix et Rhizoma | + |
| 59 | MOL005318 | Dianthramine | Ginseng Radix et Rhizoma | + |
| 60 | MOL005320 | Arachidonate | Ginseng Radix et Rhizoma | + |
| 61 | MOL005321 | Frutinone A | Ginseng Radix et Rhizoma | + |
| 62 | MOL005344 | Ginsenoside rh2 | Ginseng Radix et Rhizoma | + |
| 63 | MOL005348 | Ginsenoside-Rh4_qt | Ginseng Radix et Rhizoma | + |
| 64 | MOL005356 | Girinimbin | Ginseng Radix et Rhizoma | + |
| 65 | MOL005357 | Gomisin B | Ginseng Radix et Rhizoma | + |
| 66 | MOL005360 | Malkangunin | Ginseng Radix et Rhizoma | + |
| 67 | MOL005376 | Panaxadiol | Ginseng Radix et Rhizoma | + |
| 68 | MOL005384 | Suchilactone | Ginseng Radix et Rhizoma | + |
| 69 | MOL005399 | Alexandrin_qt | Ginseng Radix et Rhizoma | + |
| 70 | MOL005401 | Ginsenoside Rg5_qt | Ginseng Radix et Rhizoma | + |
| 71 | MOL000787 | Fumarine | Ginseng Radix et Rhizoma | + |
| 72 | MOL001406 | Crocetin | Gardeniae Fructus | + |
| 73 | MOL001663 | (4aS,6aR,6aS,6bR,8aR,10R,12aR,14bS)-10-hydroxy-2,2,6a,6b,9,9,12a-heptamethyl-1,3,4,5,6,6a,7,8,8a,10,11,12,13,14b-tetradecahydropicene-4a-carboxylic acid | Gardeniae Fructus | + |
| 74 | MOL001941 | Ammidin | Gardeniae Fructus | + |
| 75 | MOL004561 | Sudan III | Gardeniae Fructus | + |
| 76 | MOL000358 | β-sitosterol | Gardeniae Fructus | + |
| 77 | MOL001494 | Mandenol | Gardeniae Fructus | + |
| 78 | MOL001506 | Supraene | Gardeniae Fructus | + |
| 79 | MOL001942 | Isoimperatorin | Gardeniae Fructus | + |
| 80 | MOL002883 | Ethyl oleate (NF) | Gardeniae Fructus | + |
| 81 | MOL003095 | 5-hydroxy-7-methoxy-2-(3,4,5-trimethoxyphenyl)chromone | Gardeniae Fructus | + |
| 82 | MOL007245 | 3-Methylkempferol | Gardeniae Fructus | + |
| 83 | MOL009038 | GBGB | Gardeniae Fructus | + |
| 84 | - | Hyodeoxycholic Acid | Suis Fellis Pulvis | - |
| 85 | - | Glycohyodeoxycholic acid | Suis Fellis Pulvis | - |
| 86 | - | Glycochenodeoxycholic acid | Suis Fellis Pulvis | - |
| 87 | - | Taurohyocholic acid | Suis Fellis Pulvis | - |
| 88 | - | Taurohyodeoxycholic acid | Suis Fellis Pulvis | - |
| 89 | - | Taurochenodeoxycholic acid | Suis Fellis Pulvis | - |
| 90 | - | Chenodeoxycholic acid | Suis Fellis Pulvis | - |
| 91 | - | Tauroursodeoxycholic acid | Suis Fellis Pulvis | - |
| 92 | - | Deoxycholic acid | Suis Fellis Pulvis | - |
| 93 | - | Glycohyocholic Acid | Suis Fellis Pulvis | - |
| 94 | - | Taurocholic acid | Suis Fellis Pulvis | - |
| 95 | - | Taurolithocholic acid | Suis Fellis Pulvis | - |
| 96 | - | Cholic acid | Suis Fellis Pulvis | - |
| 97 | - | Lithocholic acid | Suis Fellis Pulvis | - |
| 98 | - | Hyocholic acid | Suis Fellis Pulvis | - |
| 99 | - | Bilirubin | Suis Fellis Pulvis | - |
| 100 | - | Glycocholic acid | Suis Fellis Pulvis | - |
